# Immune development differs between preterm newborns fed mothers’ own milk and donor milk

**DOI:** 10.1016/j.isci.2025.112918

**Published:** 2025-06-17

**Authors:** Ziyang Tan, Wen Zhong, Hanna Danielsson, Aron Arzoomand, Tadepally Lakshmikanth, Qi Chen, Jaromir Mikes, Jun Wang, Yang Chen, Anna James, Anders K. Nilsson, Anders Elfvin, Nele Brusselaers, Theo Portlock, Pia Lundgren, Karin Sävman, Dirk Wackernagel, Ingrid Hansen-Pupp, David Ley, Mathias Uhlén, Ann Hellström, Petter Brodin

**Affiliations:** 1 Unit for Clinical Pediatrics, Department of Women’s and Children’s Health, Karolinska Institutet, 17165 Solna, Sweden; 2 Science for Life Laboratory, Department of Biomedical and Clinical Sciences (BKV), Linköping University, Linköping, Sweden; 3 Centre for Translational Microbiome Research, Department of Microbiology, Tumor and Cell Biology, Karolinska Institutet, Stockholm, Sweden; 4 Sach’s Children’s and Youth Hospital, Södersjukhuset, Stockholm, Sweden; 5 The Sahlgrenska Centre for Pediatric Ophthalmology Research, Department of Clinical Neuroscience, Institute of Neuroscience and Physiology, Sahlgrenska Academy, University of Gothenburg, Gothenburg, Sweden; 6 Department of Pediatrics, Institute of Clinical Sciences, University of Gothenburg Sahlgrenska Academy, Gothenburg, Sweden; 7 Region Västra Götaland, Department of Neonatology, The Queen Silvia Children’s Hospital, Sahlgrenska University Hospital, Gothenburg, Sweden; 8 Global Health Institute, Department of Family Medicine and Population Health, University of Antwerp, Antwerp, Belgium; 9 Department of Ophthalmology, Sahlgrenska University Hospital, Region Västra Götaland, Gothenburg, Sweden; 10 Karolinska University Hospital, Astrid Lindgren’s Children’s Hospital, Neonatal Department, Stockholm, Sweden; 11 Karolinska Institutet, Department of Clinical Science, Intervention and Technology (CLINTEC), Stockholm, Sweden; 12 Lund University, Skåne University Hospital, Department of Clinical Sciences, Lund, Pediatrics, Lund, Sweden; 13 Department of Neuroscience, Karolinska Institutet, Stockholm, Sweden; 14 Science for Life Laboratory, Department of Protein Science, KTH - Royal Institute of Technology, Stockholm, Sweden; 15 Department of Immunology and Inflammation, Imperial College London, W12 0NN London, UK; 16 Medical Research Council Laboratory of Medical Sciences (LMS), Imperial College Hammersmith Campus, London, UK

**Keywords:** Immunology, Female reproductive endocrinology

## Abstract

Extremely preterm infants are at risk of immune-mediated complications such as infections and inflammatory conditions like bronchopulmonary dysplasia and necrotizing enterocolitis. Preterm infants are immunologically distinct from term infants at birth, but subsequently undergo adaptive postnatal changes resulting in immunological convergence during their first 3 months. Here, we performed a systems-level analysis of immune development in 72 preterm infants born as early as 22 weeks to investigate factors associated with variation. We find similar immune trajectories during early postnatal immune development but occurring more slowly in infants born at 22–24 weeks. Immune development showed a greater resemblance to that of term-born children in preterm infants fed mother’s own milk compared to donor milk. This developmental normalization was manifested by NK cell development and was not explained by differences in microbial colonization between feeding groups, possibly suggesting direct effects of bioactive milk molecules on developing immune cells in extremely preterm infants.

## Highlights

• Infants at 22–24 weeks show slower immune development than those born closer to term
• Mother’s milk exposure creates immune profiles more like full-term infants
• Mother’s milk benefits involve bioactive molecules, not just microbiome effects
• Birth timing and early nutrition influence immune maturation in very preterm babies

## Graphical abstract

**Figure:**
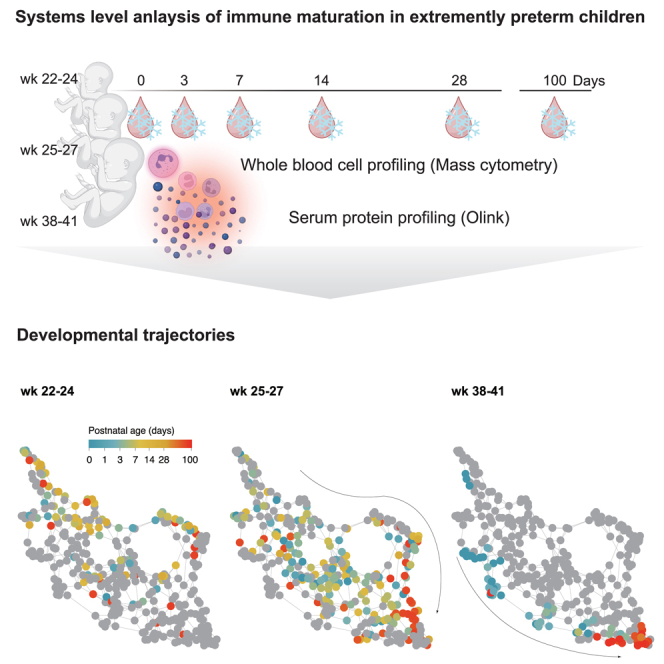

## Introduction

Preterm birth accounts for 35% of all newborn deaths.^1^ Infection and its related complications are significant contributors to death and morbidity in these infants. The immune system is crucial in protecting from infections; however, its development and function in preterm infants as compared to term-delivered children is not fully understood.^2^ Some studies have indicated that the immune system in preterm infants is less developed as compared to term-delivered children at birth, making them more susceptible to infections.^3^^,^^4^^,^^5^ Immune system development in preterm infants is a complex process influenced by various factors, including gestational age, birth weight, and environmental factors.^5^ Studies in preterm infants have indicated deficiencies in both innate and adaptive immunity, as these systems mainly develop during the third trimester of pregnancy,^2^^,^^6^ a period spent outside the womb for the most prematurely delivered infants. Therefore, understanding the immune system development of preterm infants and providing safe methods to optimize immune cell adaptation to postnatal life could render promising strategies for preventing infections and improving outcomes in this vulnerable population.

Breast milk is widely recognized as providing infants with numerous benefits, not only due to its comprehensive nutritional profile^7^^,^^8^ but also its content of antibodies, cytokines, growth factors, and other immune factors that help support and enhance immune defense and guide healthy immune system development.^7^^,^^9^^,^^10^ However, the interaction between breast milk and systemic immune cell development is poorly understood and merits further exploration. We have previously shown that human milk oligosaccharides are degraded by specific microbes in the newborn gut and promote the growth of the same strains, which in turn produce metabolites that impact differentiating T cells and potentially other immune cells as well.^11^

In this study, we analyze samples from children in a recent clinical trial of fatty acid supplementation in preterm children to explore the longitudinal developmental changes of innate and adaptive immune systems and the role of different sources of nutritional support, specifically mother’s own milk or donor milk. These sources differ with respect to several factors including pasteurization, storage conditions, and personalized adaptation (infant gestational age, time of day, and changing nutritional requirements). Immunological benefits were found for mother’s own breast milk that differed from pasteurized donor milk, which could not be explained by differences in microbiome colonization. We believe this analysis offers valuable insights and directions for future exploration of bioactive molecules present in milk which could improve immune cell development preterm infants.

## Results

### Systems-level immunomonitoring in extremely preterm infants

**Figure 1:**
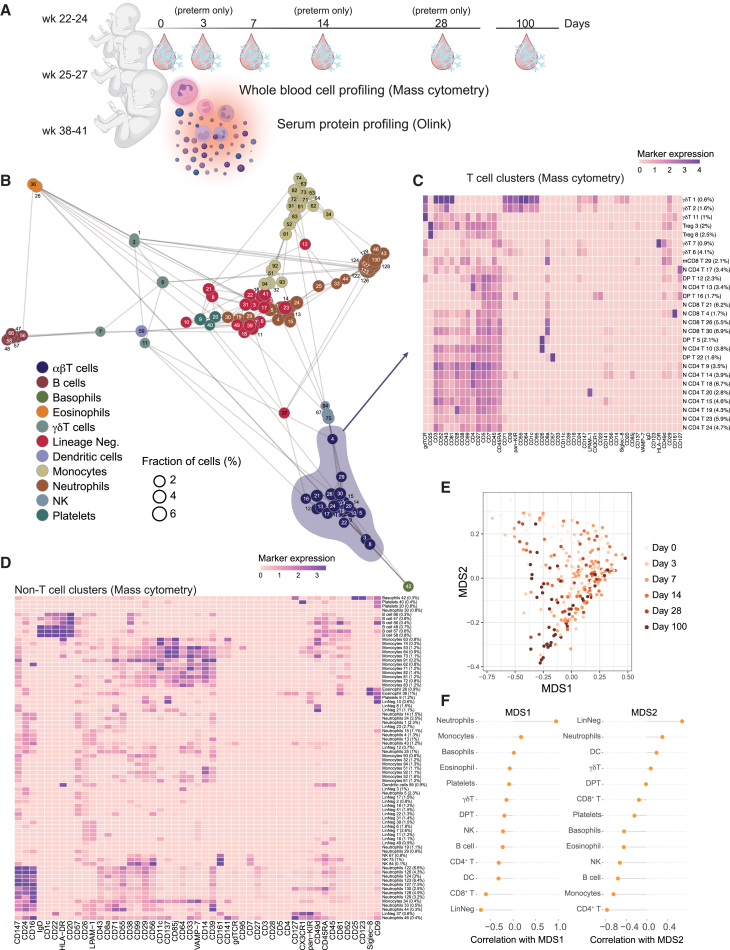
Immune landscape of extremely preterm infants and term infants as controls (A) Cohort of preterm and term infants monitored by collection and preservation of small volume blood samples at birth, days 0, 3, 7, 14, 28 and 100 (preterm) and days 0, 7, 100 (term children). Samples were frozen directly at blood draw using Cytodelics whole blood cell stabilizer and subsequently analyzed by mass cytometry while pure serum was frozen and analyzed by Olink (538 proteins). (B) FlowSOM clustering of *n* = 14322499 cells from all samples (*n* = 291) based on mass cytometry results and plotted using a force directed graph and colored by manually assigned immune subpopulation. (C) T cell clusters combined, and relevant marker expression (cluster median) shown in a heatmap. (D) Clusters of non-T cells combined, and relevant marker expression (cluster median) shown in a heatmap. (E) Longitudinal trend of immune subpopulation changes for extremely preterm infants visualized by multidimensional scaling (MDS) (*n* = 291 samples). (F) Correlation between MDS1/2 and immune subpopulation frequencies.

To investigate immune development in extremely preterm infants born before 28 weeks of gestation, we performed a secondary analysis based on the multicenter randomized clinical trial Mega Donna Mega (NCT03201588).^12^ We obtained longitudinal blood cells from 75 infants and serum proteins from 182 infants in this cohort but focused on the 72 individuals with overlapping samples covering both data types. Results of 64 longitudinal samples from 25 term infants were from a previous study was included for reference.^5^ The preterm infants were sampled on postnatal days 0, 3, 7, 14, 28, and 100 (Figures 1A and S1). Infants in the group weeks 22–24 were born at gestational age 22 weeks 0 days (22 + 0) to 24 weeks 6 days (24 + 6), while infants in the group weeks 25–27 were born at gestational age 25 weeks 0 days (25 + 0) to 27 weeks 6 days (27 + 6) (Table S1).

Mass cytometry was performed using a panel of 49 markers resulting in high-quality data from 14,322,499 single cells. These cells were subjected to FlowSOM clustering^13^ and embedded in a force-directed graph to illustrate cluster similarities (Figure 1B) and were manually annotated based on median marker expression (Figures 1C and 1D). T cells (*n* = 2,262,597) from preterm infants are highly diverse, as illustrated by median marker expression across 30 clusters determined as T cells in a second iteration of FlowSOM analyses for increased resolution (Figure 1C). As expected,^1^ the fraction of T cells with reduced CD45RA expression, indicative of memory differentiation, was very low in these newborn infants (Figure 1C). T cells expressing the γδT cell receptor constitute a larger relative fraction in newborn immune systems (8.2% of all T cells) than in adults (∼5% of all T cells^14^), emphasizing their important roles in early life immunity.^15^^,^^16^ A similar trend was also observed in Tregs, which are important for ensuring fetal-maternal tolerance *in utero* but also establishment of beneficial immune-microbe interactions after birth^17^ (Figure 1C).

The postnatal longitudinal changes in immune cell composition were heterogeneous, but followed a general trend as illustrated by a multidimensional scaling (MDS) of pairwise distances between samples revealing a gradient correlated with postnatal age at sampling (Figure 1E). The changes in MDS1 were mainly associated with decreasing neutrophil and increasing CD8^+^ T cell proportions, whereas the increase in CD4^+^ T cells, monocytes, B cells, and NK cells after birth were best captured by MDS2 (Figure 1F).

Within the neutrophil lineage we observed high expression of CD147 (extracellular matrix metalloproteinase inducer or EMMPRIN), a mediator of the inflammatory responses^18^ (Figure 1D).

### Immune system differences at birth in infants of different gestational ages

The extent to which intrauterine development and postnatal environmental exposure contribute to shaping the early-life immune system remains controversial. Many key features of the immune system are believed to develop mainly in the third trimester; however, substantial B cell diversity is observed as early as gestational week 12,^6^ while maternal antibodies can offer important protection from infections with a repertoire that was recently found to be less different in preterm as compared to term infants than historically reported.^19^

**Figure 2:**
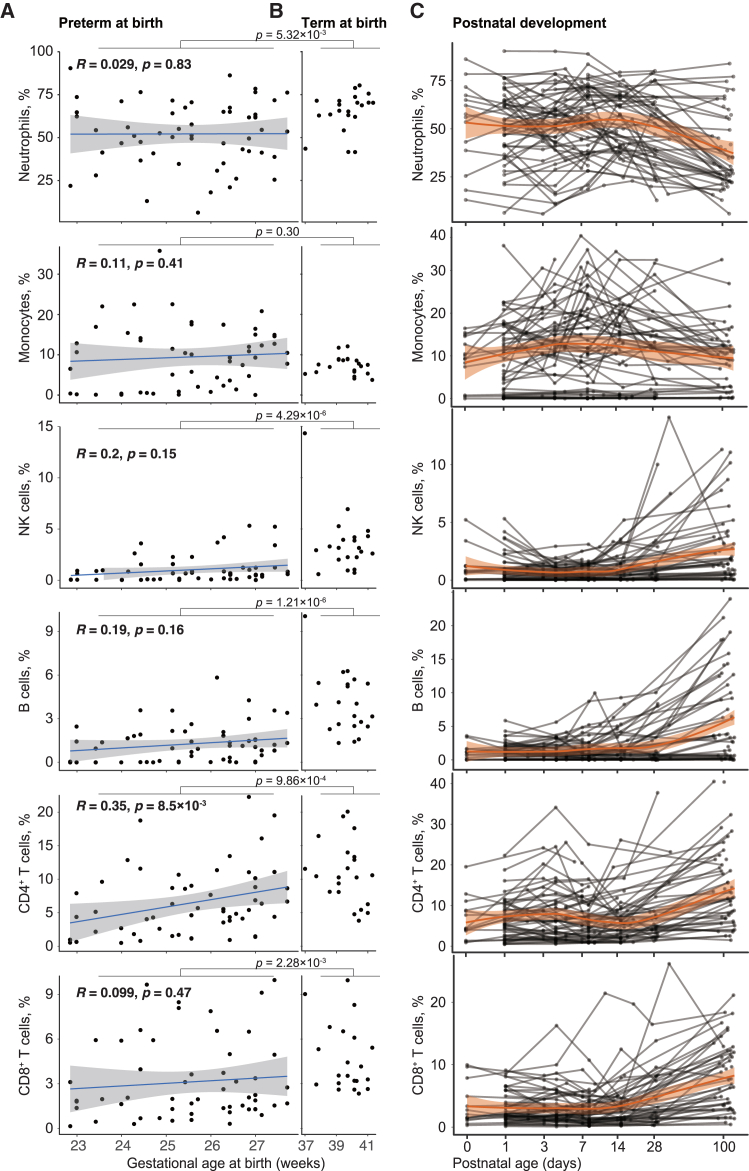
Gestational age at birth and immune cell frequencies (A) Individual cell population frequencies measured by mass cytometry at the time of birth in extremely preterm infants (*n* = 75). Linear regression analysis of correlation between fraction of cells and gestational age at birth for six major cell lineages. Shaded area represents 95% confidence interval. (B) same as in (A) but for term infants (*n* = 25) using the same Y axis scale. Wilcoxon test is used to compare preterm vs. term infants at birth. *p*-values are adjusted using BH method. (C) Preterm infants exhibit postnatal developmental changes in the same six cell lineage abundances. Trendline generated by LOESS. Shaded area represents 95% confidence interval.

Here, we examined the immune cell population frequencies and their correlation with gestational age (weeks) at birth. The immune system at birth is minimally influenced by environmental exposure; therefore, by comparing the immune systems of infants of different gestational ages at birth, we aimed to describe intrauterine immune maturation. We found that the fraction of CD4^+^ T cells increased significantly with gestational age at birth, whereas other immune cells did not show a relationship with age (Figure 2A). Within the increasing fraction of CD4^+^ T cells, the subset composition was highly heterogeneous among the individuals (Figure S2A). Simultaneously, the fraction of lineage negative cells (stem and progenitor cells), dendritic cells and γδT cells decreased with gestational age at birth (Figure S2B). In infants born at 27 weeks of age, the level of CD4^+^ T cells was comparable to that of full-term newborns, whereas the levels were lower among more immature infants (Figure 2B). Such developmental changes during fetal life could contribute to the different susceptibilities of extremely preterm infants to infections compared to more mature infants, who also have a lower risk of complications.

By comparing the immune cell frequencies between extremely preterm and term infants, we inferred that the development of CD4^+^ Ts cell levels occur gradually from the end of the second trimester (23 weeks) to term. In comparison, NK and B cells mainly increase during the third trimester (29–40 weeks). However, prenatal changes in immune cell populations were not as drastic as those observed during the first 100 days of postnatal life (Figure 2C). After birth, the developmental changes that occur among preterm infants across all gestational ages are variable but follow similar trajectories. Similarly, Kamdar et al. reported the ability of extremely preterm infants (<28 weeks) to rapidly acquire adult-like immune functionality after birth.^20^ Adaptive lymphocytes, B and T cells, both increase during the first three months of postnatal life indicative of adaptive responses to commensal microbes and other environmental exposures seen after birth.

### Longitudinal monitoring of serum protein profiles of extremely preterm infants

**Figure 3:**
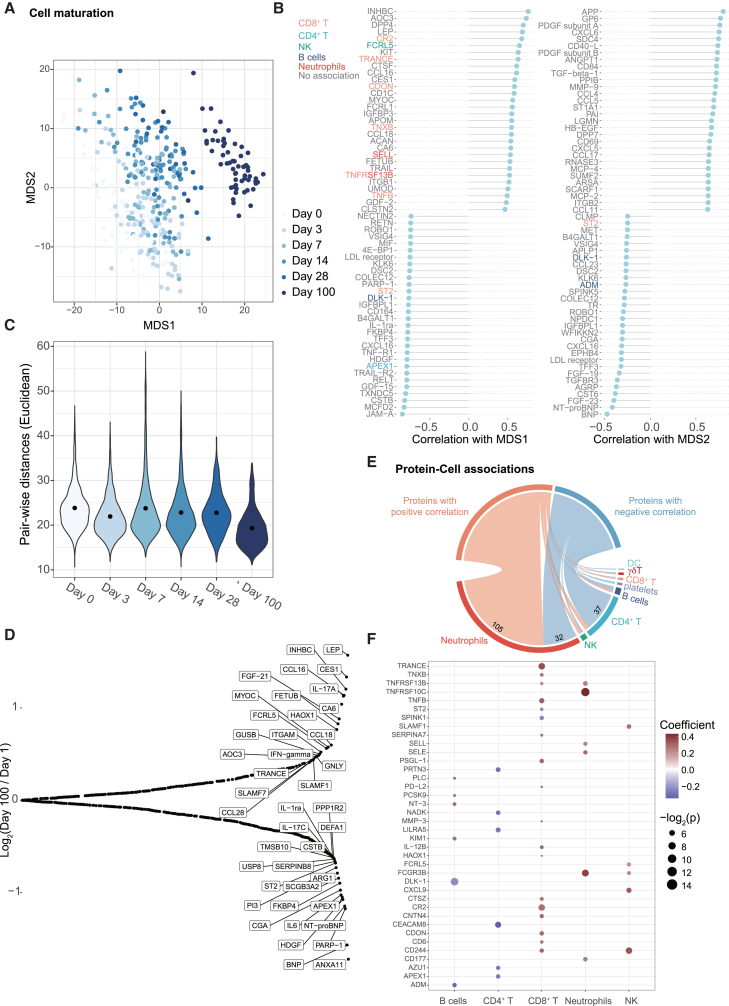
Serum protein profiles of extremely preterm infants (A) Longitudinal trend of serum protein changes during the first 100 days of life visualized by MDS (*n* = 371 samples). (B) Top correlations between MDS1/2 and serum protein levels. The proteins whose levels are explained by immune subpopulation frequencies in f are colored accordingly. (C) Inter-individual distances (Euclidean) of serum protein profiles across the timepoints. (D) Log_2_ fold change between the median serum protein levels in samples from day 100 and day 1. (E) Correlations between immune subpopulation frequencies and serum protein levels. The lower part shows the number of proteins with positive/negative correlations with selected subpop. (F) Coefficients and adjusted *p*-values of immune subpopulations in linear mixed effect models explaining the serum protein levels. In the mixed effect models, subpopulation frequencies were used as fixed effects while the intercept was allowed to vary for each child and the day of life. Details are explained in *Method*.

To further study immune system development in these preterm infants, we monitored serum protein levels longitudinally using proximity extension assays (Olink). Six Olink panels were used in the study, including Cardiometabolic, Cardiovascular II, Cardiovascular III, Development, Inflammation, and Metabolism, resulting in 552 protein assays and 541 unique proteins. Classical MDS, also known as principal coordinate analysis (PCoA), demonstrated a progressive change in serum protein profiles over time (Figure 3A). The longitudinal changes in serum protein profiles were captured by MDS1 from negative to positive. The top MDS1-correlated proteins are shown in Figure 3B. The levels of specific proteins can be further explained by the immune subpopulation levels in mixed-effect models, as described later (Figures 3B and 3F). Certain proteins may also be related to other aspects of infant development, such as nutrition, where one example is leptin (LEP), a peptide hormone released from adipose cells and associated with body weight.^21^ Individual heterogeneity was observed during the first four weeks, followed by a decrease in pairwise distances and convergence in serum protein profiles at day 100 (Figure 3C), as we previously reported in a different cohort.^5^ Compared to serum protein profiles at birth, extremely preterm infants had higher levels of both nutritional markers (LEP, FGF-21^22^) and immune markers on day 100. Higher levels of IFNγ (Th1 cytokine), TRANCE (T cell differentiation^23^), CCL16 (secreted by IFNγ-stimulated monocytes), and GNLY (cytotoxicity) were also seen at day 100 (Figure 3D).

To examine the relationship between serum protein profiles and immune subpopulations, we performed a linear correlation analysis (Figure 3E) and show that 105 proteins were positively correlated with neutrophil levels, whereas 32 were negatively correlated. As we also observed a postnatal decrease in neutrophil levels (Figure 2C), we wondered whether the observed correlations were driven by postnatal days of life after birth. That is, for example, both neutrophil levels and protein levels decreasing due to time lapse can have strong positive correlation. Therefore, we performed mixed-effect modeling using linear regression but allowed the intercept to vary by days of life and individual. Using this approach, we aimed to alleviate the influences of interindividual heterogeneity and days of life, without overly dissecting the dataset. Proteins with significant correlations with any immune subpopulation are shown in Figure 3F. The observed associations between neutrophils level and TNFRSF10C, SELL, and CD177 have also been reported in the human protein atlas.^24^ In adaptive lymphocytes, the level of CD8^+^ T cells had a positive contribution to the levels of various immune-related proteins except for ST2, SPINK1, and MMP-3, while the significant contribution from the level of CD4^+^ T cells to the protein levels were all negative.

### Developmental immune system changes in the extremely preterm differ from those of more mature preterm and term infants

**Figure 4:**
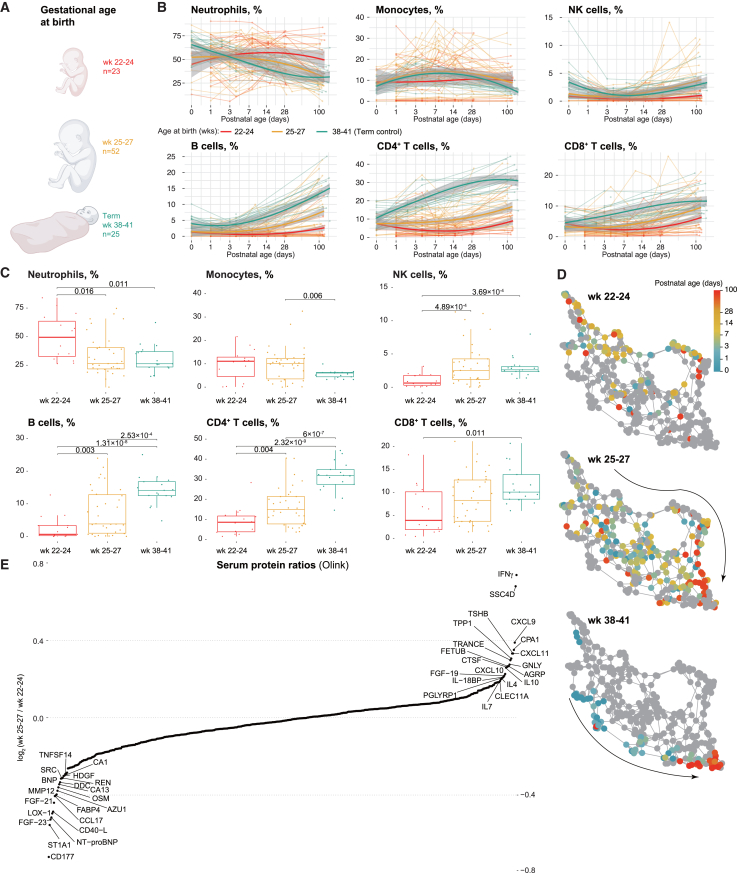
Postnatal immune development differs among groups of extremely preterm infants according to gestational age (A) Groups of infants to be compared with respect to immune development. (B) Postnatal developmental changes of immune cell population frequencies across the three groups of infants. Trendlines are obtained by LOESS with CI 95% shown as gray shades (C) Statistical comparisons and FDR adjusted *p*-values and boxplots of indicated immune cell populations at 100 days after birth. Boxplots show median (center line), interquartile range (box), and whiskers extending to 1.5× IQR. (D) K-Nearest Neighbor (kNN) (k = 4) graphs with Fruchterman-Reingold layout generated from immune subpopulation frequencies. Each node is a sample, and the color represents the postnatal age when the sample was collected. Gray nodes are the nodes belonging to other gestational age groups. (E) Log_2_(fold-change) of median protein levels (538 proteins) measured by Olink assays at 100 days of postnatal life comparing infants born weeks 25–27 vs. weeks 22–24.

Although we found that immune cell levels at birth (except for CD4^+^ T) were independent of gestational age in the extremely preterm population, (Figure 2A), the most immature infants born at gestational weeks 22–24 are at the highest risk of complications and mortality.^25^ We grouped the infants into extremely preterm children born in gestational weeks 22–24, extremely preterm born in weeks 25–27, and term infants from a different cohort^5^^,^^11^ (Figure 4A).

Surprisingly, although the two extremely preterm groups of infants exhibited few differences in immune cell composition at birth, normalization of immune development was found to occur at different rates (Figure 4B). Infants in the weeks 25–27 group showed immune system development closer to that of term controls and achieved comparable levels of neutrophils and NK cells at around 100 days, while the weeks 22–24 population showed much slower development. Regarding B cells and T cells, the differing rates are clear, with term controls being the fastest, followed by the weeks 25–27 group and then the weeks 22–24 group (Figures 4B and 4C). These differences still exist when we split the infants by their inflammatory complications such as bronchopulmonary dysplasia (BPD) and necrotizing enterocolitis (NEC) (Figures S3A and S3B). An illustration of the rate of immune development was generated by constructing a k-nearest neighbor (kNN) graph using immune cell frequencies (Figure 4D). The samples of term infants have a uniform and clear path longitudinally, and this trend is also shared by samples of weeks 25–27 infants, although with a wider distribution. However, no clear path was observed in the samples of weeks 22–24 infants.

Comparison of serum protein profiles between infants in the two groups at day 100 (Figure 4E) revealed higher levels of both a Th1 cytokine (IFNγ), Th1 differentiation regulators (CXCL9, -10, −11),^26^ and Th2 cytokines (IL4, IL10) in the weeks 25–27 group, which infers stronger adaptive immune responses in these infants.^27^ The higher levels of MMP12 in the weeks 22–24 group could be associated with the elevated CD147 in neutrophils (Figure 1D), known to induce matrix metalloproteinases as mentioned, thereby contributing to enhanced inflammatory effects in these immature infants.

### Premature infants fed mothers’ own milk show greater normalization of immune development compared to those fed donor milk

Postnatal immune development is influenced by feeding practices.^28^ Human milk feeding is associated with protection against various complications of preterm birth and reduces the risk of later diseases such as allergies, asthma, and autoimmune conditions.^29^ The mechanisms of these protective effects are not fully understood but likely involve beneficial effects on early life immune-microbe interactions with the promotion of beneficial microbes that metabolize the human-milk oligosaccharides present in human milk.^11^ Even less understood is the extent to which the immunomodulatory effect of milk is personalized and tailored to a particular infant by its mother. To investigate this further, we compared postnatal immune development in extremely preterm infants (*n* = 70) exposed to variable amounts of either mothers’ own milk or pasteurized donor milk obtained from unrelated donors.

**Figure 5:**
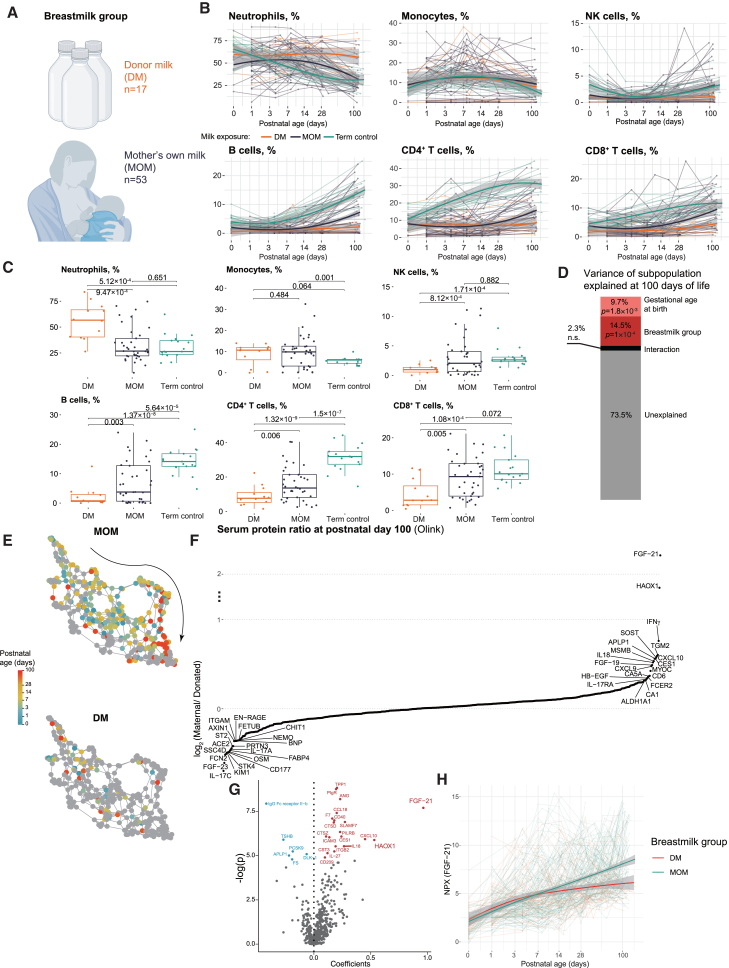
Postnatal immune development partially normalized by mother’s own milk in extremely preterm infants (A) Preterm infants were fed with a mixture of mother’s own milk and donor milk, the DM MOM were classified by the proportion of mother’s own milk, with <20% as DM group and ≥20% as MOM group. (B) Frequencies of key cell lineages in relation to days of life. Samples from each baby are connected by lines and colored by maternal milk conditions. Trendlines are obtained by LOESS with CI 95% shown as gray shades. (C) The frequency differences between maternal milk groups at 100 days of life. The *p*-values are adjusted by FDR. Boxplots show median (center line), interquartile range (box), and whiskers extending to 1.5× IQR. (D) PERMANOVA test to explain the variance in cell subpopulations measured at postnatal day 100 caused by gestational age at birth group and maternal milk group and unexplained variance. The *p*-values are provided on the stacked bars. (E) The same KNN graph as in Figure 4D but split by milk exposure. (F) 538 proteins measured at postnatal day 100 in MOM and DM infants. (G) Mixed effect model to examine effects of fraction of mother’s own milk on individual protein levels. A positive coefficient (red) indicates the positive correlation between fraction of mother’s own milk and protein level. (H) The longitudinal changes in FGF-21 level in DM and MOM groups.

In this study, most infants investigated were receiving mixtures of their mother’s own milk and donor milk due to various clinical conditions affecting both mothers and infants. Initially, infants were grouped into three groups according to the proportion of mother’s own milk they received in the first 28 days, with 0–20% as the donor milk group, 20–80% as the mixed group, and 80–100% as the mother’s milk group (Figures S4A and S4B). In a preliminary analysis, no statistical differences were observed between the mother’s milk group and the mixed group in any of the investigated immune subpopulations at 100 days of life after birth (Figure S4C). Weak Spearman correlations were observed when taking a fraction of mother’s own milk as a continuous variable (Figure S4D). Therefore, we merged these two groups to increase statistical power and dissected the cohort into two human milk groups, the mother’s own milk group (MOM, 20–100% of mother’s own milk) and the donor milk group (DM, 0–20% of mother’s own milk) (Figure 5A).

Infants in both MOM and DM groups followed similar postnatal immune development trajectories, and both groups showed delayed expansions of B cells and T cells after birth (Figure 5B). This delayed lymphocyte expansion was more pronounced in children receiving donor milk during their first 100 days (Figure 5B). When compared 100 days after birth proportions of neutrophils, NK cells, B cells, CD4^+^ T cells, and CD8^+^ cells in the blood differed significantly between DM and MOM fed preterm (Figure 5C). These differences still existed when the inflammatory complications of the infants were taken into consideration (Figures S5A and S5B). To examine whether these differences were driven by gestational age as previously described, we performed a permutational analysis of variance (PERMANOVA) test for the immune cell composition at 100 postnatal days (Figure 5D). We found that gestational age and milk feeding significantly explained 9.7% and 14.5% of the variance, respectively. The remaining variance (73.5%) was unexplained by either factor and possibly arises from interindividual heterogeneity and inflammatory conditions. A kNN graph was then used to assess the delayed postnatal immune development in the MOM and DM groups (Figure 5E). A longitudinal trend was observed in MOM group but not the DM group. A reference trend from term infants is illustrated in Figure 4D. When investigating serum protein profiles (Figure 5F), we found elevated FGF-21 levels in MOM infants, which could indicate regulated energy homeostasis,^22^ which has been reported to be associated with mother’s milk.^30^ MOM infants also have higher levels of a Th1 cytokine (IFNγ), Th1 differentiation regulators (CXCL9, -10), and an IFNγ inducer (IL18), while Th2 response cytokines (IL4 and IL10) were absent. These results suggest a skewed Th1 response in MOM infants, which is described to be promoted by breastfeeding and *Bifidobacteria* colonization.^11^

To further understand the relationship between serum protein profiles and human milk, we constructed linear mixed-effects models between the fraction of the mother’s own milk and individual protein levels (Figure 5G). Again, FGF-21 was strongly associated with the mother’s own milk, as was HAOX1, an oxidase involved in fatty acid metabolism in the liver.^31^ Levels of FGF-21 also differed longitudinally between the MOM and DM groups (Figure 5H), indicating its potential as a biomarker of breastmilk intake since the amount of milk ingested is often difficult to assess in small infants and weight gain is used as an imperfect surrogate marker that is often confounded by several other factors. However, few differences in immune cell composition emerged when comparing FGF-21-high and low infants longitudinally (data not shown).

### Gut microbiome colonization at 28 days and 34 weeks in relation to milk intake

**Figure 6:**
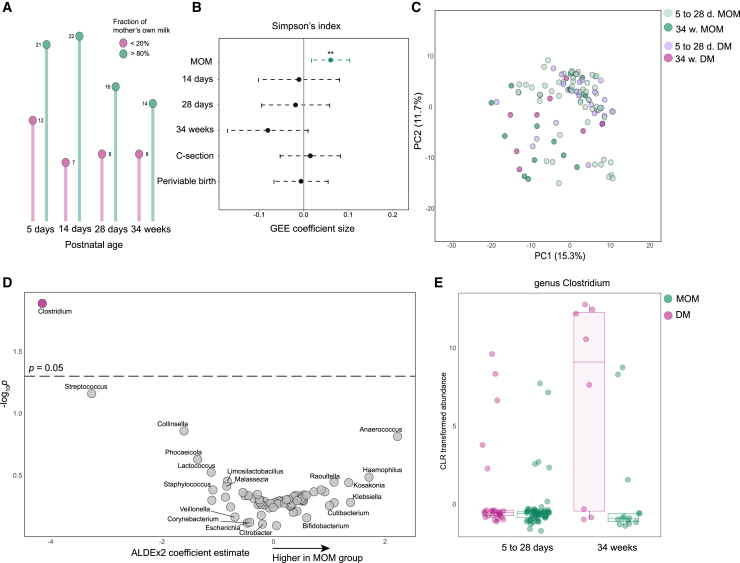
Comparison of gut microbiomes in DM and MOM infants (A) Description of the cohort of the microbiome analysis. Sample numbers are shown for each group. All infants in the cohort were born between 22 and 28 weeks of gestation. (B) Generalized estimating equation (GEE) model to explain the alpha diversity (Simpson’s index) of each sample by breastmilk groups (DM or MOM), postnatal age, mode of delivery and gestational age at birth. Positive coefficients indicate positive correlation between alpha diversity and the listed covariates. ∗*p* < 0.05, ∗∗*p* < 0.01. (C) Principal component analysis of microbiome compositions using Aitchison’s distance. Samples are colored by time-point and breastmilk group. (D) Results of ANOVA-like differential expression (ALDEx2). Positive coefficients indicate positive correlation with MOM feeding. Y axis shows uncorrected *p* values. (E) Abundances of genus Clostridium from 5 to 28 days and at 34 weeks of MOM and DM samples. The abundances are transformed by centered log-ratio transformation (CLR).

Previous studies have associated breastfeeding with the selection for bacteria able to metabolize its nutritional components such as human milk oligosaccharides, leading to immunomodulatory effects associated with health benefits.^11^^,^^32^^,^^33^ To assess the gut microbial compositional differences between infants fed with DM or MOM, we performed metagenomic sequencing of stool samples collected within one month of birth and at 34 weeks from 61 extremely preterm infants in the Mega Donna Mega trial cohort mentioned above. DM was defined >80% DM intake, and MOM >80% MOM intake (Figure 6A). Simpson’s index was the main alpha diversity (diversity within sample) metric that differed between the groups, poised toward a slightly more even microbiome in infants fed with MOM (Figure 6B). This difference could not be explained by sequencing batch (Figure S6G). Across time for both groups, the diversity metric Simpson’s index decreased in parallel with an increase in richness, likely due to continuous microbial colonization from birth (Figures S6A–S6C). Principal component analysis showed larger distances within the 34-week samples compared to the samples from 5 to 28 days (Figure 6C). Few differences were observable between the DM and MOM fed infants for both timepoints. Gut microbial variation was predominantly explained by time and individuality, and to a lesser extent by the delivery mode (Figures S6E and S6F). When testing 5 to 28 days or 34-week samples separately, MOM did not significantly explain any variation.

We then performed differential abundance testing with ALDEx2^34^ using count data and generalized estimating equations on both relative abundances and centered log-ratio (CLR) transformed data (Figure S6H). At the genus level, the only significantly different taxa across the three methods were Clostridium, which primarily had higher abundance in DM fed infants at 34 weeks (Figures 6D, 6E, and S6H). This is consistent with previous literature.^35^ At 34 weeks, no species differed significantly between the groups using ALDEx2. However, several Staphylococcus, Streptococcus, and Clostridium species appeared to be biased toward DM fed infants (Figure S6I). These results indicate that differences in microbial colonization of the intestine as measured by stool metagenomics cannot fully explain the differences in immune development between children fed donor and mothers’ own milk although some potentially important strains were found to differ in abundance.

### NK cell maturation in preterm infants correlate with mothers’ own vs. donor milk

**Figure 7:**
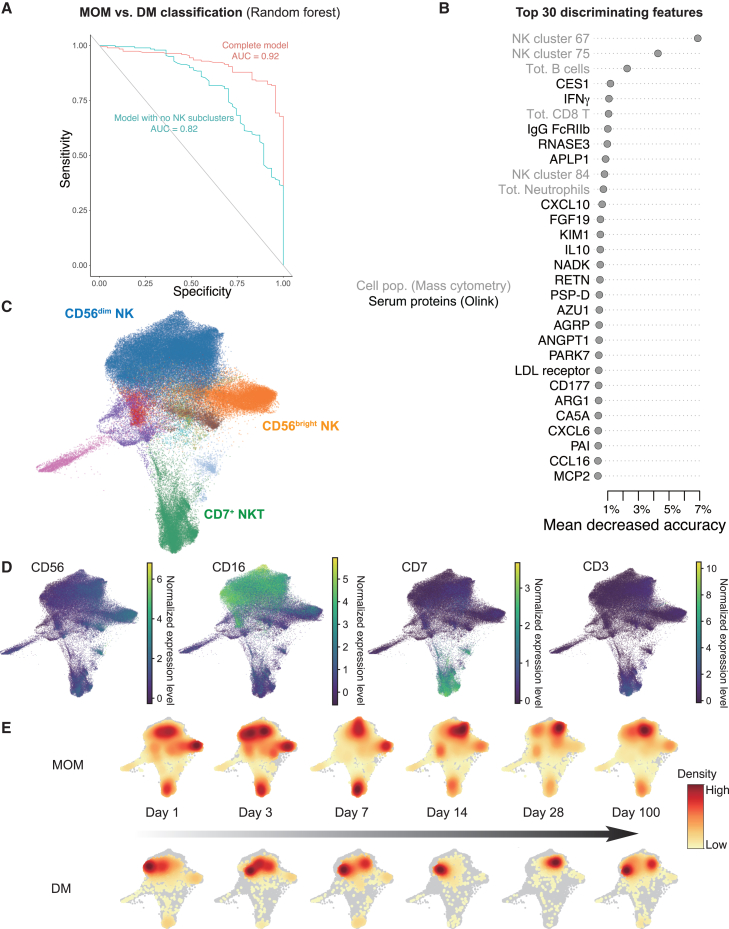
NK cell development differs between extremely preterm infants fed mother’s own milk or donor milk (A) ROC curve of random forest classifier discriminating the MOM and DM groups. Repeated cross validation (repeats = 3, folds = 5) was used during the training. The AUCs of two models are presented in the figure. (B) Top discriminatory features from random forest classifier colored by feature type. The x axis represents the decrease of accuracy of the classifier if the feature is excluded. (C) Single NK cell embedding (*n* = 93311 cells) and clustering (Leiden) from mass cytometry data of all samples (270 samples from 72 infants). (D) Key NK cell markers distinguishing clusters in (C). (E) PAGA embedding showing cellular densities of NK cells projected onto the same PAGA embedding as in (C-D) across timepoints in infants receiving mother’s own milk and donor milk respectively.

Given the trends of variable immune development between infants fed MOM and DM, we sought to identify the most divergent immune system features underlying these differences. We performed random forest classification and found differentiation between MOM and DM groups. This classification was based on serum proteins (Olink analyses), immune cell clusters, and broad immune cell lineages (meta clusters), as well as clinical features such as postnatal days and gestational age groups in Figure 4. The receiver operating characteristic (ROC) curve showed an area under the curve (AUC) of 0.92 (Figure 7A). We performed feature selection analysis and present the top 30 features distinguishing MOM and DM groups (Figure 7B). NK cell clusters 67, 75, and 84 (as well as total NK cell abundance (STAR Methods) were among the top features with higher abundances in the MOM group (Figure 7B). After removing NK clusters from the random forest classifier, the AUC dropped from 0.92 to 0.82 confirming their importance (Figure 7A).

NK cells are critical during fetal life, required for efficient placentation, and involved in feto-maternal tolerance.^36^^,^^37^ Also, these innate cells are important components of newborn immune systems during viral infections and the CD56^bright^ subset of NK cells exert immunoregulatory functions.^38^ NK-22 cells have been reported to produce IL-22 and contribute to tolerance at mucosal sites.^39^ To assess individual NK cell phenotypes in preterm infants and differences in relation to milk exposure, we performed partition-based graph abstraction (PAGA)^40^ of single cell data (mass cytometry). Clusters are generated by 49 markers with the Leiden algorithm, colored accordingly, and then embedded with ForceAtlas2.^41^ Three clusters are manually annotated according to 4 key markers (Figures 7C and 7D). When separated by time point and milk intake, we found that CD56^dim^CD16^+^ NK cells dominate in DM children, while a substantial fraction of immunoregulatory CD56^bright^CD16^dim^ NK cells were found in the MOM group (Figure 7E). These findings suggest a possible impact of bioactive molecules in mothers’ own, but not donor milk with possible implications for intestinal colonization and a previously overlooked mechanism of regulation of inflammatory responses via breastmilk.

## Discussion

Extremely preterm infants (born <28 weeks of gestation) are incredibly vulnerable due to anatomical, clinical, and environmental factors. Preterm birth occurs for a reason which often involves an infection or an inflammatory disease in the pregnant mother, such as preeclampsia. The risks are greatest for the smallest infants with 1-year survival rates of infants born in gestational week 22 at around 30%, although a striking improvement has been achieved during the most recent decade.^42^ Severe inflammatory complications like NEC and BPD are now less frequent because of improved care, while intraventricular hemorrhages and retinopathy of prematurity (ROP) have remained stable and occurring at similar rates in extremely preterm infants, despite improvements in overall survival.^42^ To reduce the incidences of ROP in preterm infants, a randomized controlled trial (Mega Donna Mega) was launched in Swedish centers in 2016 to test the use of enteral arachidonic acid (AA) and docosahexaenoic acid (DHA), previously shown to be deficient in preterm infants^43^ and important enablers of vascular recovery following hypoxia-induced retinal damages.^44^ The trial found that AA/DHA-supplemented preterm infants had higher serum levels of these fatty acids and a 50% reduced incidence of severe ROP but no effect on the incidences of NEC, BPD, or other inflammatory complications.^12^ In this study, we perform additional analyses of the infants taking part in this trial and focus on immune system development. We find no differences among AA/DHA supplemented groups on immune cell abundances. Instead, we unravel important differences among infants born at the extreme end of preterm delivery (22–24 weeks) as compared to the slightly more mature group (25–27 weeks) as compared to term controls. In line with our previous results, we see dramatic postnatal changes in immune cell abundances and serum proteins, likely driven by environmental exposures such as colonizing microbes.^5^ The groups converge over time after birth as immune developmental changes occur similarly across groups and the differences seen at the time of birth are reduced.

Here, we also uncover a difference when comparing infants fed mother’s own milk as compared to human donor milk, where immune development is accelerated and converges toward healthy term infant trajectories in those receiving MOM. This is intriguing as donor milk use in extremely preterm infants is common. Donor milk is most often compared to infant formula feeding and is associated with reduced risks of NEC, shorter time on parenteral nutrition, but also slower growth rates and longer time to recover birth weight as compared to formula feeding.^45^ We are mindful of the fact that not all mothers can or want to breastfeed due to maternal medication use or other reasons. We believe it is important to better understand the bioactive molecules conferring these effects such that better supplements can be developed and allow for beneficial effects to be provided for all newborn children.

The donor milk used in Swedish NICUs is treated at 62.5°C for 30min (Holder pasteurization) to kill possible pathogenic microbes but obviously also affects possible commensals similarly. Enzymes and live immune cells present in the donor milk are also destroyed by Holder pasteurization while most macronutrients remain intact.^46^ Molecules important for microbiome colonization and establishment of immune-microbe interactions such as lactoferrin^47^ and EGF^48^ are reduced in Holder pasteurized donor milk as are IgA antibodies important not only as protectors of mucosal membranes from infections but also necessary for anchoring of beneficial microbes in the mucus layer and promoting their colonization.^49^ An additional difference between MOM and DM is that milk donors typically donate milk when their infants are several months old while MOM is provided by mothers who just delivered a preterm child and since milk composition changes throughout lactation, this could contribute to compositional differences. The relative contribution of either of these factors or other lesser-known mediators of the beneficial effects of mother’s own over donor milk on immune development cannot be determined in this cohort study but should be further explored in animal model systems.

We have previously reported that human milk oligosaccharides (HMOs) promote beneficial microbes equipped for the metabolism of HMOs leading to the production of metabolites of immunomodulatory potential such as Th1 skewing, and upregulation of inhibitory molecules for tolerance.^11^ Note that the control of feeding practices was only in the first 28 days, but differences were observed thereafter, indicating a long-lasting effect of initial breastfeeding and mother’s own milk. Another important observation made is that specific bacteria, predominantly a Clostridial species were overrepresented in infants obtaining donor milk. It is possible that in the absence of MOM, lack of beneficial commensals promoted by milk IgA anchoring leads to a microbiome where other species seize an opportunity to proliferate and grow. Aggregated, many of these taxa belong to the genus Clostridium, however, at the species level no individual taxa were differentially abundant. This indicates that many taxa can expand and that these taxa are variable within the DM group, while the MOM group is more homogeneous, indicating the selectivity of promoting effects by IgA and other factors lost upon pasteurization. The association between milk feeding and NK cell composition and abundance is intriguing and in line with previous results showing effects of transplacental IgG antibodies impacting NK cells via Fc-receptor binding,^50^ as well as a role for bioactive human milk lactoferrin in modulating NK cell function.^51^ More modest differences might also be present although a larger sample size is likely required to detect these. Cumulatively, from this cohort there is limited evidence of larger gut microbial compositional differences playing a role in the immunological differences observed between DM and MOM-fed infants.

In conclusion, we find distinct changes in extremely preterm infants’ immune systems during the first three months of life, that are influenced by gestational age at birth, but also environmental factors postnatally. Infants fed mother’s own milk display stronger effects on developing immune cells and epithelia, leading to a more rapid convergence to the healthy trajectory of term infants. Ultimately, our findings suggest that critical elements could be added back to donor milk to improve its immunological benefits such as bioactive molecules lost during pasteurization, or antibodies mismatched between mothers and their offspring thus failing to target colonizing microbes, factors which could represent a missed opportunity for healthy immune system development early in life.

### Limitations of the study

Any study of extremely ill and preterm children who receive intensive care for weeks to months following their delivery will be highly complex and standardized inclusion, care, or sampling will be impossible. Here we have enrolled extremely preterm children undergoing neonatal intensive care and compared children born at different gestational ages and fed different nutritional supplements. We have tried our best to balance groups among comparisons, but as shown in Table S1, some differences among groups persist and are explained by different care requirements among children born preterm at different gestational ages and experiencing different complications. The feeding groups are not completely balanced for all other parameters such as mode of delivery or gestational age, but this is impossible to achieve in a clinical study of rare cases such as this one and conclusions must be drawn with these complexities in mind. Another limitation of this study is the focus on cell frequencies and phenotypes and not functional responses, but given the complexity of obtaining longitudinal small volume samples we were unable to conduct any stimulation experiments. Presenting cell frequencies instead of absolute cell numbers could also be a shortcoming. Another limitation is the relatively small number of patients studied, but extreme prematurity is fortunately relatively rare and conducting longitudinal sampling of such critically ill patients undergoing intensive neonatal care is highly complicated both ethically, logistically, and practically and thus limited our inclusion rates. Replication of findings will require additional cohorts and was beyond the scope of this study. This is another important limitation.

## Resource availability

### Lead contact

Further information and requests for resources and reagents should be directed to and will be fulfilled by the Lead Contact, Petter Brodin (petter.brodin@ki.se).

### Materials availability

This study did not generate new unique reagents.

### Data and code availability

• All Data are shared through Mendeley Data and the DOI is included in the key resources table.
• All codes required to reproduce the figures and analyses are available via our dedicated GitHub repository: https://github.com/Brodinlab/preterm_DHA.
• Any additional information involved in this paper is available from the lead contact upon request.

## Acknowledgments

The Brodin laboratory is supported by 10.13039/501100000781European Research Council (ERC StG-), HORIZON-HLTH-2022-STAYHLTH-02 grant agreement 101094099 (INITIALISE), EASI-Genomics (ID: 824110), the 10.13039/501100004359Swedish Research Council (2019-01495, 2020-06190, 2020-02889, 2021-06529, 2021-05450, 2022-01567), and 10.13039/501100004063Knut and Alice Wallenberg Foundation (KAW2023-0344, VC-2021-0026, KAW 2020.0102), 10.13039/100016408Göran Gustafsson Foundation (GG2020¬0040), 10.13039/501100003748Swedish Society for Medical Research (SSMF, CG- 22-0148-H-02 to P.B.) and 10.13039/501100004047Karolinska Institutet (2019-01019, 2018-02229). We thank the Affinity Proteomics Facility at SciLifeLab in Stockholm for conducting the Olink analyses and the nurses at Sahlgrenska University Hospital, Karolinska University Hospital, and Lund University Hospital for sample and data collection. We acknowledge the entire Human Protein Atlas program, the Science for Life Laboratory and the Centre for Translational Microbiome Research for their valuable contributions. We also thank Catharina Löfqvist for sample handling. The computational analyses were enabled by resources at Uppsala Multidisciplinary Center for Advanced Computational Science (UPPMAX). Additional funding was provided by the Erling Persson Foundation, the Swedish Research Council (#2016-01131, #2022-01562 to A.H.), SciLifeLab & Wallenberg Data Driven Life Science Program (KAW 2020.0239). Government grants under the ALF agreements (ALFGBG-717971, ALFGBG-971188 and ALFGBG-812951 to A.H.), the 10.13039/501100005689Gothenburg Medical Society, Frimurare Barnhusdirektionen, 10.13039/501100011831De Blindas Vänner, 10.13039/100018666Carmen and Bertil Regnér's Foundation, Ögonfonden, Cronqvist’s foundation, the Wallenberg Clinical Scholars (to A.H.), LillaBarnetsFond and Swedish infant death foundation. H.D. was supported by Region Stockholm. Z.T. gratefully acknowledges financial support from 10.13039/501100004543China Scholarship Council.

## Author contributions

Z.T. analyzed most of the data except for Olink data, analyzed by Q.C., W.Z., and Z.T. and metagenomics data analyzed by A.A., with input from P.B., T.P., and N.B. Clinical data collection was led by H.D., A.H., and A.E. Patient enrollment and clinical data collection by D.L., I.H.-P., D.W., K.S., P.L., A.K.N., and A.E. Cytometry data was generated by T.L. Blood processing protocol by J.M., T.L., and J.W. Database for generated lab data by Y.C. Study design by A.H. and M.U. and immunological analysis plan by P.B. Paper written by Z.T., A.J., and P.B.

## Declaration of interests

P.B., T.L., and J.M. are cofounders of Cytodelics AB (Stockholm, Sweden) which produce and distribute whole blood cell stabilizer solutions used within this study. P.B. is an executive board member of Kancera AB, scientific advisor for Pixelgen Technologies AB, Helaina Inc., Scailyte AG, Oxford Immune Algorithmics Ltd, Sention Health AB, and the Swedish Olympic committee.

## STAR★Methods

### Key resources table

**Table:** 

REAGENT or RESOURCE	SOURCE	IDENTIFIER
**Antibodies**		

Anti-human CD1c (L161), Purified	BioLegend	Cat# 331502; RRID: AB_1088995
Anti-human CD3e (UCHT1), Purified	BioLegend	Cat# 300401; RRID: AB_314055
Anti-human CD4 (RPA-T4), Purified	BioLegend	Cat# 300502; RRID: AB_314070
Anti-human CD5 (UCHT2), Purified	BioLegend	Cat# 300602; RRID: AB_314088
Anti-human CD7 (CD7-6B7), Purified	BioLegend	Cat# 343102; RRID: AB_1659214
Anti-human CD8 (SK1), Purified	BioLegend	Cat# 344702; RRID: AB_1877104
Anti-human CD9 (SN4 C3-3A2), Purified	eBiosciences	Cat# 14-0098-82; RRID: AB_657777
Anti-human CD11c (Bu15), Purified	BioLegend	Cat# 337202; RRID: AB_1236381
Anti-human CD14 (M5E2), Purified	BioLegend	Cat# 301802; RRID: AB_314184
Anti-human CD16 (3G8), Bi-209	Standard BioTools	Cat# 3209002B, RRID: AB_2756431
Anti-human CD20 (2H7), Purified	BioLegend	Cat# 302302; RRID: AB_314250
Anti-human CD22 (HIB22), Purified	BioLegend	Cat# 302502; RRID: AB_314264
Anti-human CD24 (ML5), Purified	BioLegend	Cat# 311102; RRID: AB_314851
Anti-human CD25 (2A3), Sm-149	Standard BioTools	Cat# 3149010B, RRID: AB_2756416
Anti-human CD26 (BA5b), Purified	BioLegend	Cat# 302702; RRID: AB_314286
Anti-human CD27 (L128), Er-167	Standard BioTools	Cat# 3167006B; RRID: AB_2738042
Anti-human CD28 (CD28.2), Purified	BioLegend	Cat# 302902; RRID: AB_314304
Anti-human CD29 (TS2/16), Purified	BioLegend	Cat# 303002; RRID: AB_314318
Anti-human CD33 (WM53), Purified	BioLegend	Cat# 303402; RRID: AB_314346
Anti-human CD38 (HIT2), Purified	BioLegend	Cat# 303502; RRID: AB_314354
Anti-human CD39 (A1), Purified	BioLegend	Cat# 328202; RRID: AB_940438
Anti-human CD43 (84-3C1), Purified	eBiosciences	Cat# **14-0439-82**; RRID: AB_11219080
Anti-human CD45 (HI30), Y-89	Standard BioTools	Cat# 3089003B; RRID: AB_2661851
Anti-human CD45RA (HI100), Tm-169	Standard BioTools	Cat# 3169008B; RRID: AB_394678
Anti-human CD49d (9F10), Pr-141	Standard BioTools	Cat# 3141004B; RRID: AB_467292
Anti-human CD52 (HI186), Purified	BioLegend	Cat# 316002; RRID: AB_389275
Anti-human CD55 (JS11), Purified	BioLegend	Cat# 311302; RRID: AB_314859
Anti-human CD56 (NCAM16.2), Purified	BD	Cat# 559043; RRID: AB_397180
Anti-human CD57 (HCD57), Purified	BioLegend	Cat# 322302; RRID: AB_535988
Anti-human CD64 (10.1), Purified	BioLegend	Cat# 305002, RRID: AB_314486
Anti-human CD71 (CY1G4), Purified	BioLegend	Cat#, 334102; RRID: AB_1134247
Anti-human CD81 (5A6), Purified	BioLegend	Cat# 349502, RRID: AB_10643417
Anti-human CD85j (GHI/75), Purified	BioLegend	Cat# 333702, RRID: AB_1089089
Anti-human CD95 (DX2), Purified	BioLegend	Cat# 305602, RRID: AB_314540
Anti-human CD99 (HCD99), Purified	BioLegend	Cat# 318002; RRID: AB_604112
Anti-human CD123 (6H6), Purified	BioLegend	Cat# 306002; RRID: AB_314576
Anti-human CD127 (A019D5), Ho-165	Standard BioTools	Cat# 3165008B; RRID: AB_2738043
Anti-human CD137/4-1BB (4B4-1), Purified	BioLegend	Cat# 309802; RRID: AB_314781
Anti-human CD141 (M80), Purified	BioLegend	Cat# 344102; RRID: AB_2201808
Anti-human CD147 (HIM6), Purified	BioLegend	Cat# 306202; RRID: AB_314586
Anti-human CD161 (HP-3G10), Purified	BioLegend	Cat# 339902; RRID: AB_2661837
Anti-human CX3CR1 (8E10.D9), Purified	BioLegend	Cat# 824001; RRID: AB_2564876
Anti-human HLA-DR (L243), Purified	BioLegend	Cat# 307602; RRID: AB_314680
Anti-human IgD (IA6-2), Purified	BioLegend	Cat# 348202; RRID: AB_10550095
Anti-human KIR2DL2/L3, Purified	BioLegend	Cat# 312602; RRID: AB_314933
Anti-human KIR2DL1/KIR2DS5, Purified	R&D Systems	Cat# MAB1844-100; RRID: 10973635
Anti-human KIR3DL2/CD158k, Purified	R&D Systems	Cat# MAB2878; RRID: AB_10829223
Anti-human LPAM-1 (Hu117), Purified	R&D Systems	Cat# MAB10078-100; RRID: AB_2927804
Anti-human Siglec-8 (837535), Purified	R&D Systems	Cat# MAB7975; RRID: AB_2927805
Anti-human TCRγδ (5A6.E9), Purified	Fisher Scientific	Cat# TCR1061; RRID: AB_223500
Anti-human VAMP-7 (549115), Purified	R&D Systems	Cat# MAB6117; RRID: AB_2927806

**Biological samples**		

Whole blood and stool samples from children in Mega Donna Mega (MDM) study & Term cohort in Karolinska University Hospital, Stockholm	NICU, Gothenburg (MDM cohort); Stockholm (Term born infants)	N/A

**Chemicals, peptides, and recombinant proteins**		

Bovine Serum Albumin	Sigma-Aldrich	Cat# A3059; RRID: N/A
Cell-ID Intercalator-Ir	Standard BioTools	Cat# 201192B; RRID: N/A
Cell-ID 20-Plex Pd Barcoding Kit	Standard BioTools	Cat# 201060; RRID: N/A
Cell staining buffer (CSB)	Standard BioTools	Cat# 201068; RRID: N/A
DMSO	Sigma-Aldrich	Cat# D8418; RRID: N/A
EDTA	Rockland	Cat# MB-014; RRID: N/A
EQ Four Element Calibration Beads	Standard BioTools	Cat# 201078; RRID: N/A
FBS	Sigma-Aldrich	Cat# 12103C; RRID: N/A
Fc Receptor (FcR) blocking buffer	Cytodelics AB	Customized in-house
Maxpar Water	Standard BioTools	Cat# 201069; RRID; N/A
Maxpar X8 Multimetal Labeling Kit (40 rxn)	Standard BioTools	Cat# 201300; RRID; N/A
Metal isotopes as chloride salts (In-115, Gd-155, Gd-157, Dy-161, Dy-163, Yb-173)	Trace Sciences International	Customized order
Paraformaldehyde	VWR	Cat# 16005; RRID: N/A
Penicillin-streptomycin	Sigma-Aldrich	Cat# P4333; RRID: N/A
Protein Stabilizer PBS	Candor Bioscience	Cat# 131125, RRID: N/A
PBS 1X	Rockland	Cat# MB-008; RRID: N/A
RPMI 1640 medium	Sigma-Aldrich	Cat# R848; RRID: N/A
Sodium Azide	Sigma-Aldrich	Cat# 71289; RRID: N/A
Whole blood (human) processing kit	Cytodelics AB	Cat# hC001-500; RRID: N/A

**Critical commercial assays**		

Cardiometabolic	Olink AB	N/A
Cardiovascular II	Olink AB	N/A
Cardiovascular III	Olink AB	N/A
Development	Olink AB	N/A
Inflammation	Olink AB	N/A
Metabolism	Olink AB	N/A

**Deposited data**		

Processed and raw data	This paper	https://doi.org/10.17632/fpc6ypbsts

**Other**		

BenchBot robot	Agilent technologies	Customized
Bravo liquid handling platform	Agilent technologies	Customized
CyTOF XT mass cytometer	Standard BioTools	N/A
EL406 Washer Dispenser	BioTek	Customized
pluriStrainer Mini, 40μm	pluriSelect	Cat# 43-10040-70; RRID: N/A
Polypropylene tubes	Sarstedt	Cat# 55526; RRID: N/A
TC20 automated cell counter	BioRad	N/A
Cellaca MX automated cell counter	Nexcelom Bioscience	N7A
Vspin microplate centrifuge	Agilent technologies	Customized

**Software and algorithms**		

CyTOF software (v. 6.0.626)	NA	https://www.standardbio.com
R v4.2.3	NA	https://www.r-project.org
premessa v0.3.4	NA	https://github.com/ParkerICI/premessa
CellGrid v0.6.1	Chen et al.^52^	https://github.com/Brodinlab/cellgrid
FlowSOM v2.2.0	Gassen et al.^13^	https://github.com/SofieVG/FlowSOM
vite v0.4.10	NA	https://github.com/ParkerICI/vite
ggraph v2.1.0	NA	https://CRAN.R-project.org/package=ggraph
lme4	Bates et al.^53^	https://cran.r-project.org/web/packages/lme4/index.html
fsmb	NA	https://cran.r-project.org/web/packages/fmsb/index.html
circlize	Gu et al.^54^	https://cran.r-project.org/web/packages/circlize/index.html
snakemake	Köster et al.^55^	https://github.com/snakemake/snakemake
StaG-mwc v0.7.0	Boulund et al.^56^	https://github.com/ctmrbio/stag-mwc
kraken2 v2.1.2	Wood et al.^57^	https://github.com/DerrickWood/kraken2
bowtie2 v2.3.1	Langmead et al.^58^	https://github.com/BenLangmead/bowtie2
MetaPhlAn4 v4.0.6	Beghini et al.^59^	https://github.com/biobakery/MetaPhlAn
fastP v0.23.2	Chen et al.^60^	https://github.com/OpenGene/fastp
mia v1.11.2	Ernst et al.^61^	https://bioconductor.org/packages/release/bioc/html/mia.html
ALDEx2 v1.30.0	Fernandes et al.^34^	https://bioconductor.org/packages/release/bioc/html/ALDEx2.html
phyloseq v1.42.0	McMurdie et al.^62^	https://bioconductor.org/packages/release/bioc/html/phyloseq.html
vegan v2.6.4	NA	https://github.com/vegandevs/vegan
randomForest v4.7-1.1	Liaw et al.^63^	https://cran.r-project.org/web/packages/randomForest/index.html
ROSE v0.0-4	Lunardon et al.^64^	https://cran.r-project.org/web/packages/ROSE/index.html
caret v6.0-94	Kuhn^65^	https://cran.r-project.org/web/packages/caret/index.html
Python v3.7.7	NA	https://www.python.org
scanpy v1.6.0	Wolf et al.^66^	https://github.com/scverse/scanpy
anndata v0.7.8	Virshup et al.^67^	https://github.com/scverse/anndata

### Experimental model and study participant details

#### Cohort description

This is a secondary analysis based on the multicenter, randomized clinical trial the Mega Donna Mega Study (MDM, ClinicalTrial.gov identifier NCT03201588).^12^ The MDM study evaluated the effect of orally administered fatty acids in 206 extremely premature infants of both sexes treated at neonatal intensive care units (NICU) in Gothenburg, Stockholm and Lund, Sweden, between 2016 to 2019. The details are described elsewhere.^12^ In summary infants born before 28 weeks of gestation were randomized to receive oral supplementation with the arachidonic acid (AA) and docosahexaenoic acid (DHA) or standard care and prospectively studied from birth to postmenstrual age 40+0 weeks. Here we investigated a subset from a single site in Gothenburg to minimize the variation among children studied due to site-specific conditions and care. The term-born cohort is borrowed from our published study as the normal control of immune development.^5^ In brief, 64 blood samples were collected at day 0, day 7 and day 100 from 25 term infants (gestational age 259 days to 289 days) at the Karolinska University Hospital, Huddinge, under the regional ethical board in Stockholm, Sweden (DNR: 2009/2052 – 31/3 & 2014/921-32). The collection and preservation of samples from the term cohort is described elsewhere.^5^

#### Ethical statement

The MegaDonnaMega-study followed the Consolidated Standards of Reporting Trials (CONSORT) reporting guideline and was approved by the regional ethical board of Gothenburg. This study was conducted following the Declaration of Helsinki principles and received approval at the University of Gothenburg, regional ethical review board (Dnr 303-11, T570-15). Informed consent was obtained from all participating infants' parents or legal guardians. The study was registered at ClinicalTrials.gov (NCT03201588).

### Method details

#### Blood sample and medical data collection

The preterm blood samples were collected at birth and repeatedly up to 40 weeks post menstrual age. Sampling aimed for nine timepoints; first day of life followed by postnatal day 3, 7, 14, 28 and PMW 30, 32, 36 and 40. Whole blood was collected in serum separator tubes with clot activator and centrifuged once before storing the remaining serum at -70°C without delay. Clinical variables including growth, morbidities and treatments were collected according to the MegaDonnaMega trial protocol. Nutritional regime data, including amounts of mother’s own milk and donor milk have been closely collected. In Sweden extremely, premature infants are provided breast milk; mother’s own milk or if this is not available, donated pasteurized milk.^68^ In this study, infants received a mixture of maternal milk and donor milk with the composition of maternal milk ranging from 0% to 100% until postmenstrual age 32 weeks. After this timepoint, the use of donor milk was replaced with formula when MOM was not sufficiently available.

#### Stool sample collection

Stool samples for metagenomic data analysis were collected at four post-natal time points: day 3-5, day 14, day 28 and at 34 weeks postmenstrual age (or at discharge from the neonatal ward). Stool samples were collected in sterile tubes and frozen and stored in -70 degrees Celsius without delay. Fresh stools were required for collection, no rectal swabs, hence the timespan of the first days in life.

#### Blood sample processing

Blood samples obtained from infants at multiple time points were processed as follows for mass cytometry analysis - 0.1-0.2ml of blood was mixed with an equal amount of stabilizer (Cytodelics AB), incubated for 10 min at ambient temperature and stored at -80°C until further processing. Stabilized samples were thawed, fixed, and lysed using Lysis and Wash buffers (Whole blood processing kit; Cytodelics AB).

#### Immune cell profiling by mass cytometry

Post fixation/lysis of stabilized whole blood samples, 1-2x10^6^ cells/sample were plated and cryopreserved using standard cryoprotective solution. For staining, cells were thawed at 37°C, barcoded using an automated liquid handling robotic system (Agilent technologies) using the Cell-ID 20-plex Barcoding kit (Standard BioTools Inc.) as per the manufacturer’s recommendations, and stained batch-wise after pooling. Antibodies targeting the surface antigens are listed in the key resources table, washed with Cell staining buffer (CSB) (Standard BioTools Inc.) and fixed with 2% formaldehyde, all of which performed using a custom-built liquid handling robotic platform (Mikes et al., 2019). Cells were then stained with Iridium-labeled DNA intercalator at a final concentration of 0.125 mM (MaxPar Intercalator-Ir, Standard BioTools Inc.) on the day of sample acquisition. Following washes with CSB, PBS, Cell acquisition solution (CAS) (Standard BioTools Inc.), cells were counted and diluted to 500,000 cells/ml containing 0.1X EQ Four Element Calibration Beads (Standard BioTools Inc.) and filtered through a 35mm nylon mesh. Samples were acquired on Helios mass cytometers (Standard BioTools Inc.) using CyTOF software version 6.0.626 with noise reduction, a lower convolution threshold of 200, event length limits of 10-150 pushes, a sigma value of 3, and flow rate of 0.045 ml/min.

#### Antibody labeling

The panel of monoclonal antibodies used for this study are indicated in the key resources table. Monoclonal antibodies were either purchased pre-conjugated from Standard BioTools or in purified carrier/protein-free buffer formulation from other vendors. Purified antibodies were conjugated to lanthanide metals using the MAXPAR X8 polymer conjugation kit (Standard BioTools Inc.), and cadmium metals using the MCP9 antibody labelling kit (Standard BioTools Inc.) according to the manufacturer’s protocol. Antibody concentration before and after conjugation was measured by NanoDrop 2000 spectrometer (Thermo Fischer Scientific) at 280 nm. Following conjugation of antibodies, they were diluted 1:1 in Protein Stabilizer PBS (Candor Bioscience GmbH) prior to use in experiments.

#### Serum proteome profiling

As described previously, serum samples were gently thawed on ice, centrifuged at 4°C 20 min 1500 × g, and 25 μl serum was transferred to 96-well microtiter plates. Plates (17 in total) were deep-frozen and sent on dry ice to Olink Bioscience (Uppsala, Sweden) for analysis. Serum proteins were analyzed using a multiplex proximity extension assay (PEA) technology (Olink Bioscience).^69^^,^^70^ Briefly, each kit consists of a microtiter plate for measuring 92 protein biomarkers in all 88 samples, and each well contained 96 pairs of DNA-labeled antibody probes. Longitudinal samples from everyone were allocated to the same plate to reduce batch-effects related to inter-individual variability. To minimize inter- and intra-run variation, the data were normalized using both an internal control (extension control) and an inter-plate control and then transformed using a pre-determined correction factor. This study uses six Olink panels including Cardiometabolic, Cardiovascular II, Cardiovascular III, Development, Inflammation, and Metabolism, resulting in 552 protein assays and 541 unique proteins. One microliter infant serum was used for each panel. The pre-processed data were provided in the arbitrary unit normalized protein expression (NPX) on a log2 scale, where a high NPX represents high protein concentration. Limit of detection (LOD) for each protein was defined as three standard deviations above the background. Protein panels from samples with more than 10% below LOD values were removed from the analysis. A preterm infant serum pool sample and 8 internal control samples were included on each plate for bridging and quality control. Three proteins with drastic fluctuations between visits were considered to have a problematic batch effect and were removed. After quality control, 538 unique proteins were kept for downstream analysis.

#### Metagenomics data generation

DNA extraction from the stool samples was done using a dual physical and chemical lysis protocol with the Quick-DNA Magbead Plus kit (D4082; Zymo Research, Irvine, CA, USA). The protocol is designed mainly for bacteria and archaea; fungi are rarely detected. Before extraction, the samples went through 1 min of bead- beating at 1600 rpm (ZR Bashing Bead lysis matrix— S6012; Zymo Research, Irvine, CA, USA) followed by 30 min of lysozyme solution treatment at 37°C (lysozyme recipe: 20 mM Tris–Cl, pH 8; 2 mM sodium EDTA [Tris–EDTA; Sigma-Aldrich, catalogue no. T9285]; lysozyme [Sigma-Aldrich, catalogue no. L6876-100G] to 100 mg/ml) and proteinase K at 55°C for 30 min (20 mg/ml, part of Zymo extraction kit). An automated high-throughput pipeline for human microbiome sampling was used for extraction, as previously described.^71^ Samples below 10,000 reads were discarded.

The sequencing was conducted using MGI whole-genome sequencing technology (MGI FS DNA library prep kit (1,000,013,455—MGI, Shenzhen, China) and sequencing kit (PE150 1,000,016,952; MGI)) in a DNBSEQ-T7 sequencer MGI as previously described.^72^ In total 50 ng of DNA was utilized for sequencing. All controls from the extraction phase and a negative PCR control were submitted to PCR and sequenced with the samples.

### Quantification and statistical analyses

#### Mass cytometry data pre-processing

The mass cytometry data were exported as FCS files from the CyTOF software. These files were processed for de-barcoding with the “premessa” package in R. An in-house supervised classification algorithm, “CellGrid”,^52^ was employed for initial identification of cells and non-cells. Non-cells such as beads, doublets, and debris were excluded based on CellGrid annotations. Moreover, cells exhibiting top 1% expression levels of any marker were also excluded from the analysis, except for CD123 and gdTCR to preserve important subgroups including plasmacytoid dendritic cells (pDCs) and basophils. Data were transformed using the hyperbolic sine function with a coefficient of 5 (arcsinh(X/5)). The processed dataset was then consolidated into a single FCS file for subsequent analysis.

#### Unsupervised clustering and manual assignment of immune cell subpopulation

The preprocessed mass cytometry data was further classified into subpopulations using the “FlowSOM” R package.^13^ We performed a three-step clustering process: initially, we clustered the data into 30 different groups to segregate neutrophil populations. Subsequently, within the remaining non-neutrophil data, we generated an additional 100 clusters. Another 30 clusters were generated using all the T cells to increase resolution. The results of the clustering steps were then combined. Manual annotation of the resulting clusters was performed by evaluating their median expression levels of specific markers.

#### Force-directed graph visualization of FlowSOM cell clusters

To visualize the cell clusters identified by FlowSOM, we generated a force-directed graph based on the median expression levels of each marker within the clusters. The marker expression data were first normalized using Z-score transformation to standardize the weight of different markers. Subsequently, the 'vite' R package was employed to construct the graph, applying an edge threshold of 5 to filter the connections. The resulting graph was visualized using the 'ggraph' R package, which utilizes the Fruchterman-Reingold layout algorithm. This algorithm employs a force-directed method to enhance visualization by distributing nodes evenly and optimizing edge lengths for improved clarity.

#### Quantitative analysis of immune cell frequency and serum protein level

The immune cell frequencies and serum protein levels obtained from previous steps were visualized by classical multidimensional scaling (MDS). In brief, Euclidean distances were calculated and then passed to cmdscale function in stats-package using R. The pseudo-loadings of MDS were generated by calculating the Pearson correlations between each feature (immune cell frequency or protein level) and MDS1/2. The pair-wise distances of serum protein profiles were obtained by gathering the upper triangle of the distance matrix generated by dist function in R. Permutational multivariate analysis of variance (PERMANOVA) was performed using the adonis2 function from the vegan-package. Bray-Curtis dissimilarity was used as the distance metric for PERMANOVA, and the permutation times was set to 30000. All statistical tests were performed using Student’s t test and adjusted by FDR if not specified.

#### Mixed effect model of immune cell frequency and serum protein level

The immune cell frequencies and serum protein levels were first centered with z-score transform and used to fit a linear mixed effect regression (lmer) model for each protein. In each lmer model, the abundances of B cells, CD4^+^ T cells, CD8^+^ T cells, NK cells, neutrophils and monocytes were assigned as fixed effects, and the intercept was allowed to vary for each child and postnatal days. The p values of the fixed effects were calculated using type II wald chi-square test and corrected by Benjamini-Hochberg method. The coefficients and confidence intervals of fixed effects in each model were estimated by 1000 simulations using the FEsim function in merTools package.

#### K-nearest neighbor graph visualization

The abundances of neutrophils, monocytes, NK cells, B cells, CD4^+^ T cells and CD8^+^ T cells from samples of term and preterm infants were used to construct the k-nearest neighbour (kNN) graph. While constructing the kNN graph, k was set to 4 and the Bray-Curtis dissimilarity was utilized as the distance metric. Afterwards, the force-directed layout algorithm by Fruchterman and Reingold was used for the visualization of the kNN graphs.

#### Processing of metagenomics data

144 demultiplexed fastq files generated from four flowcells were quality controlled and downstream processed via StaG-mwc v.0.7.0.^56^ First samples were trimmed, filtered, and deduplicated using fastp v.023.2.^60^ Samples were then run through two passes of host filtration. Once with kraken2^57^ against the GRcH38 database, and second with bowtie2^58^ against the chm13+Y v2.0 database.^73^ Taxonomic count and relative abundance data was then generated using MetaPhlAn 4.0.6.^59^ with the mpa_vOct22_CHOCOPhlAnSGB_202212 database using the “-t rel_ab_w_read_stats” flag.

#### Metagenomic data analyses and statistics

126 samples had >5 million reads, of these 108 samples belonged to subjects fed with >80% donor milk or >80% mother’s own milk and were kept for analysis. CLR transformed abundance data was generated per sample by imputing NANs with the smallest observable value in the dataset. Shannon entropy, Simpson’s evenness (1/DS), and Richness were calculated using mia in R.^61^ Generalized estimating equations was used to assess association of alpha diversity metrics with diet including gestational age, delivery mode, flowcell, timepoint, and subject ID as covariates.

Principal components analysis was performed on CLR transformed data using Euclidean distances, which is also known as Aitchison’s distance. Variation explained was assessed by running a permanova through the adonis2 package including the same covariates and the “terms” flag with 999 permutations.

Differential abundance on count data was performed with ALDEx2 running 256 Monte-Carlo simulations, adjusting for differences in library size. Generalized estimating equations was performed on CLR transformed and proportional relative abundance data. Models were generated for timepoint subsetted (5-28 days and 34w respectively) and all samples combined at both genus and species level. All models were run adjusting for covariates.

#### Training and optimization of random forest classifier

Serum proteins were first filtered according to their variance. High variance (>1) proteins and frequencies of cell lineage were then z-score transformed and combined with clinical features as the input data of random forest classifier. Randomly Over Sampling Examples (ROSE,^74^ n=400) was used to balance the input data and followed by a repeated cross validation (repeats=3, folds=5) training. The classifier had an ROCAUC of 0.86 and the NK cell frequency showed predominant importance. We then dissected the NK cell population into 3 flowSOM clusters (NK_67, NK_75, NK_84) from previous results for optimization. We substituted the NK cell frequency with the 3 flowSOM cluster frequencies and re-trained the classifier and the ROCAUC increased to 0.92.

#### Single cell embedding of NK cells using partition-based graph abstraction (PAGA)

The preprocessed mass cytometry results were batch corrected with combat and z-score transformed. The NK cells were then filtered out and subsampled (n=1000) per sample. A k-NN (k=10) was constructed based on the subsampled data and the clusters were detected by Leiden algorithm (resolution = 0.3). PAGA graph was then built and used as the initial position of ForceAtlas2. The density plots were then generated using the ForceAtlas2 embeddings.
