# Supplementary material for: Immune development differs between preterm newborns fed mothers’ own milk and donor milk
Source: iScience. 2025 Jun 17;28(7):112918. doi: 10.1016/j.isci.2025.112918 (PMC12266545; doi:10.1016/j.isci.2025.112918)
Supplement: Document S1. Figures S1–S6 and Table S1 [file mmc1.pdf]

iScience, Volume *28*

Supplemental information

Immune development differs between preterm newborns fed mothers' own milk and donor milk

Ziyang Tan, Wen Zhong, Hanna Danielsson, Aron Arzoomand, Tadepally Lakshmikanth, Qi Chen, Jaromir Mikes, Jun Wang, Yang Chen, Anna James, Anders K. Nilsson, Anders Elfvin, Nele Brusselaers, Theo Portlock, Pia Lundgren, Karin Sävman, Dirk Wackernagel, Ingrid Hansen-Pupp, David Ley, Mathias Uhlén, Ann Hellström, and Petter Brodin

Supplemental Figures

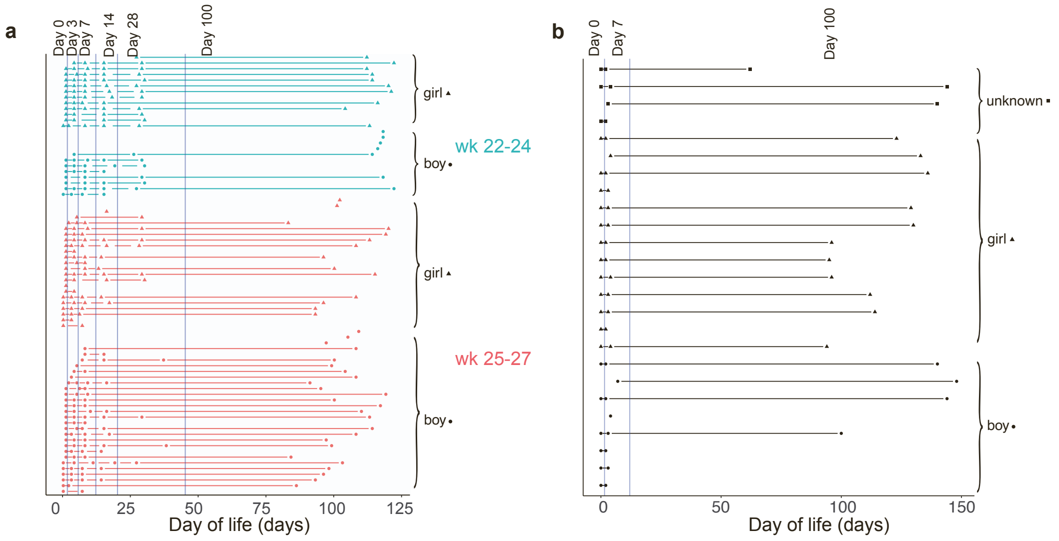

Figure S1. Overview of the cohort, Related to Figure 1. The sampling timepoint of each sample from the extremely preterm cohort (**a**) and term cohort (**b**). Samples from the same child are connected with lines. Colors and shapes of points represent the gestational age group and gender respectively.

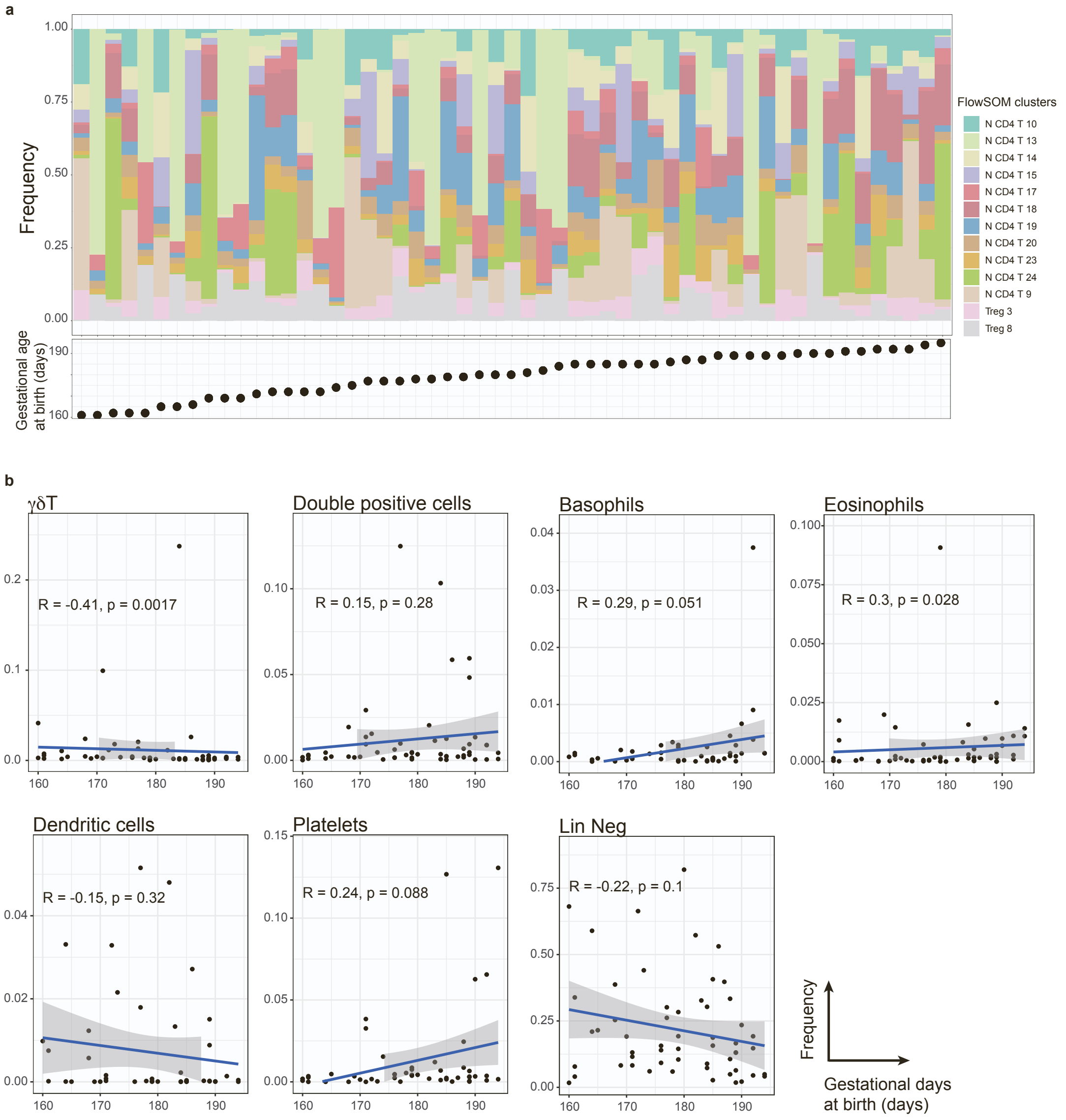

Figure S2. Immune profiles in relation to gestational age, related to Figure 2. a) The compositon of immune cell clusters generated by flowSOM within CD4$^+$ T cells at birth. **b**) Frequencies at birth of immune cell populations other than the six major ones shown in Figure 2a. Linear regression analysis of correlation between cell population fraction and gestational age at birth are shown with grey zones representing the 95% confident intervals.

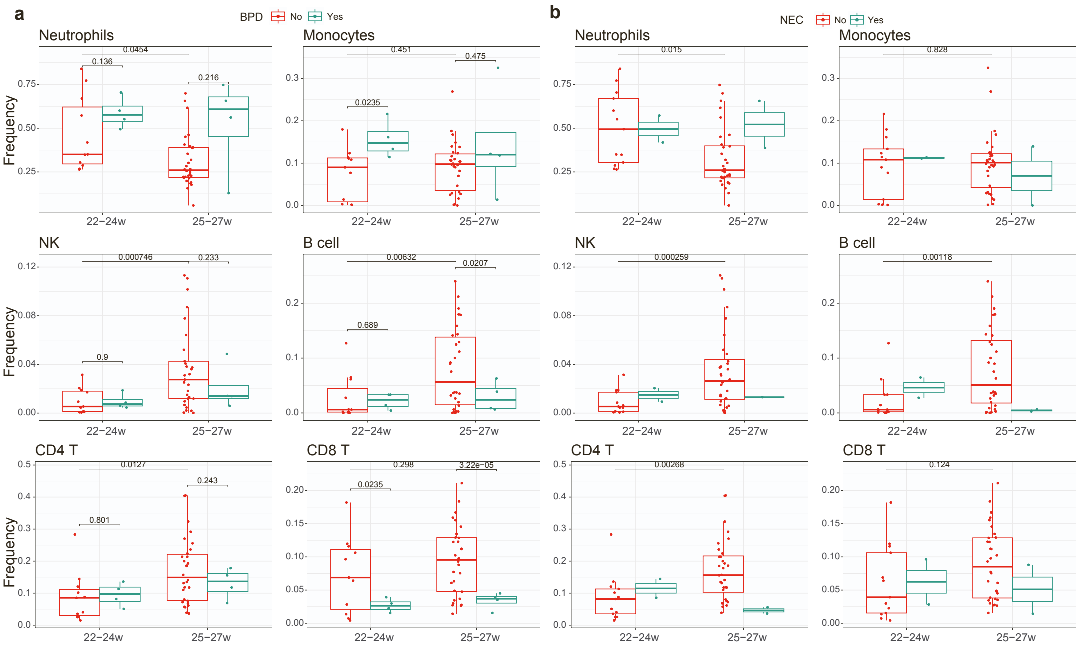

Figure S3. Immune profiles separated by gestational age and clinical conditions at birth, related to Figure 4. Subpopulation frequencies after 100 days of life of extremely preterm infants born GA weeks 22-24 or GA weeks 25-27. The boxplots are further separated by clinical conditions of (**a**) bronchopulmonary dysplasia (BPD) or (**b**) necrotizing enterocolitis (NEC). Boxplots show median (center line), interquartile range (box), and whiskers extending to 1.5× IQR.

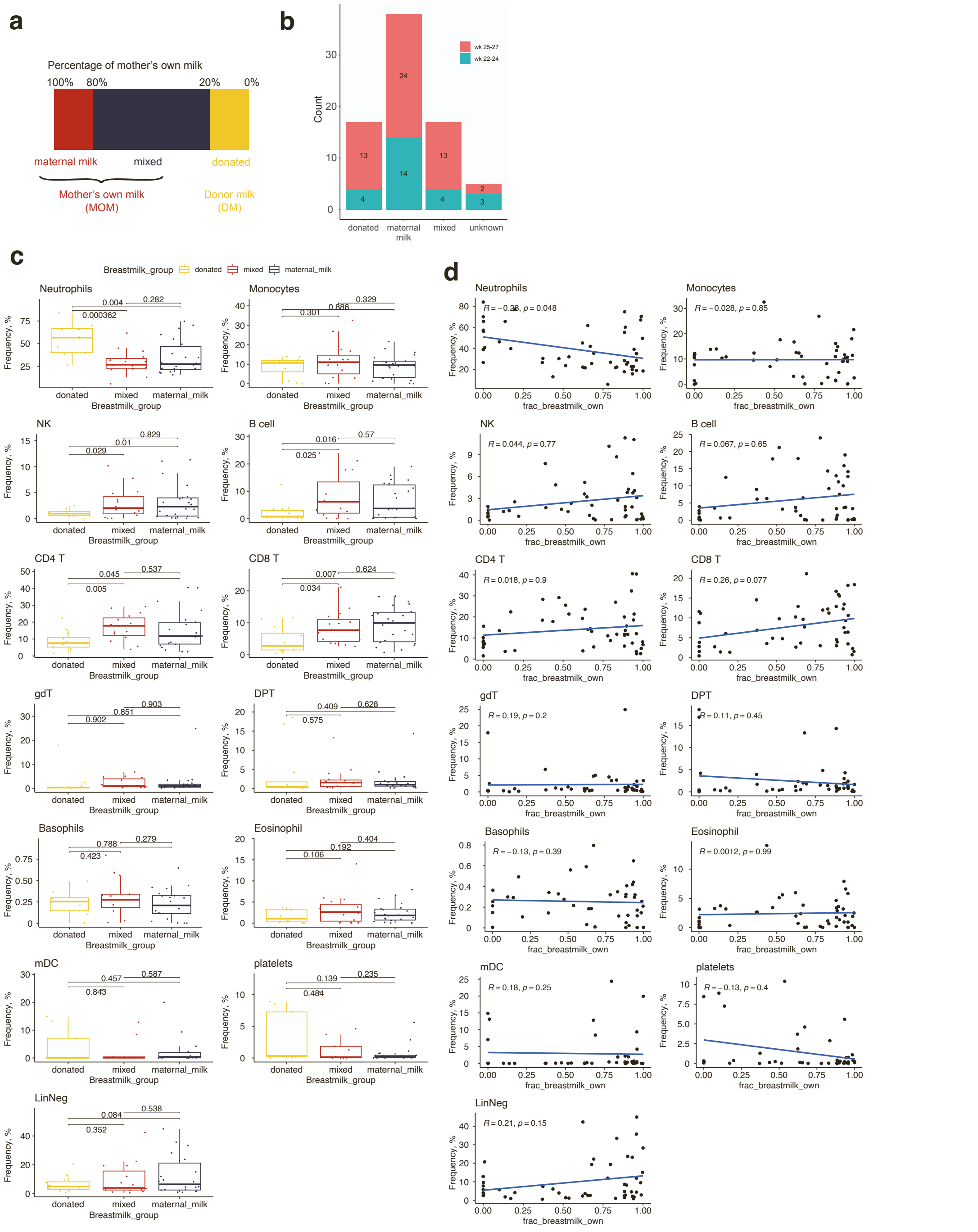

Figure S4. Subpopulation frequencies according to the fraction of maternal milk fed to the infants, related to Figure 5 a) The setup of breastmilk group according to the percentage of mother's own milk. **b**) The gestational age at birth distribution in the breastmilk groups. **c**) Adjusted *p*-values and boxplots of immune subpopulaitons at 100 days of life for the breastmilk grouping of three groups. **d**) Immune subpopulation frequencies at 100 days of life as a function of percentage of mother's own milk. R and *p*-values are from Spearman correlation. Boxplots show median (center line), interquartile range (box), and whiskers extending to 1.5× IQR.

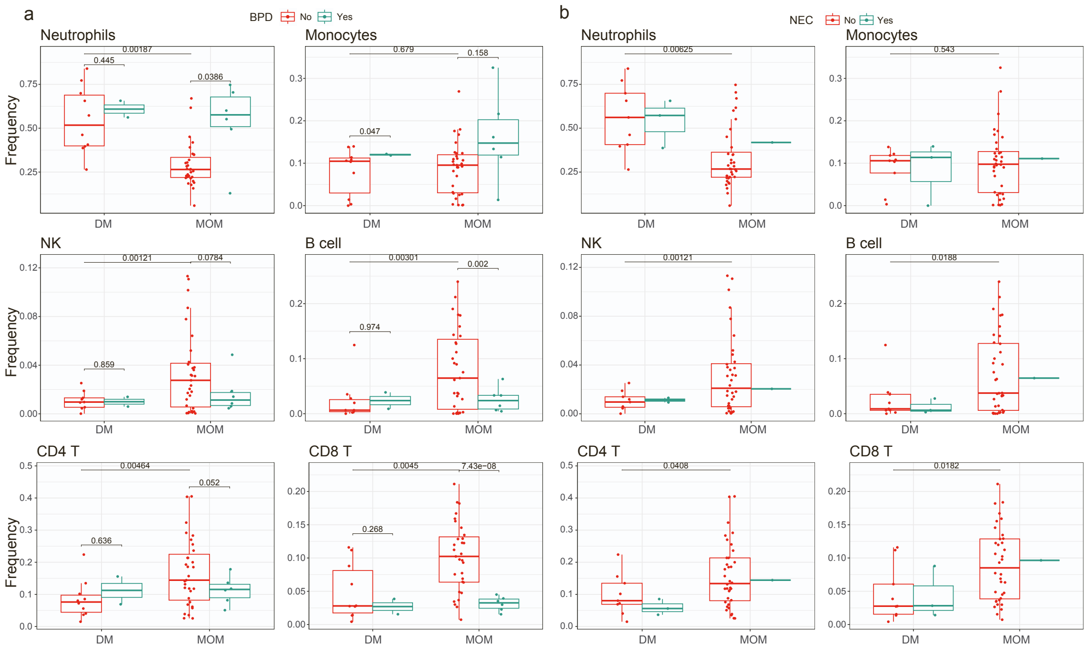

Figure S5. Immune profiles separated by DM/MOM and clinical conditions at birth, related to Figure 5. Subpopulation frequencies after 100 days of life of extremely preterm infants fed DM or MOM. The boxplots are further separated by clinical conditions of (**a**) bronchopulmonary dysplasia (BPD) or (**b**) necrotizing enterocolitis (NEC). Boxplots show median (center line), interquartile range (box), and whiskers extending to 1.5× IQR.

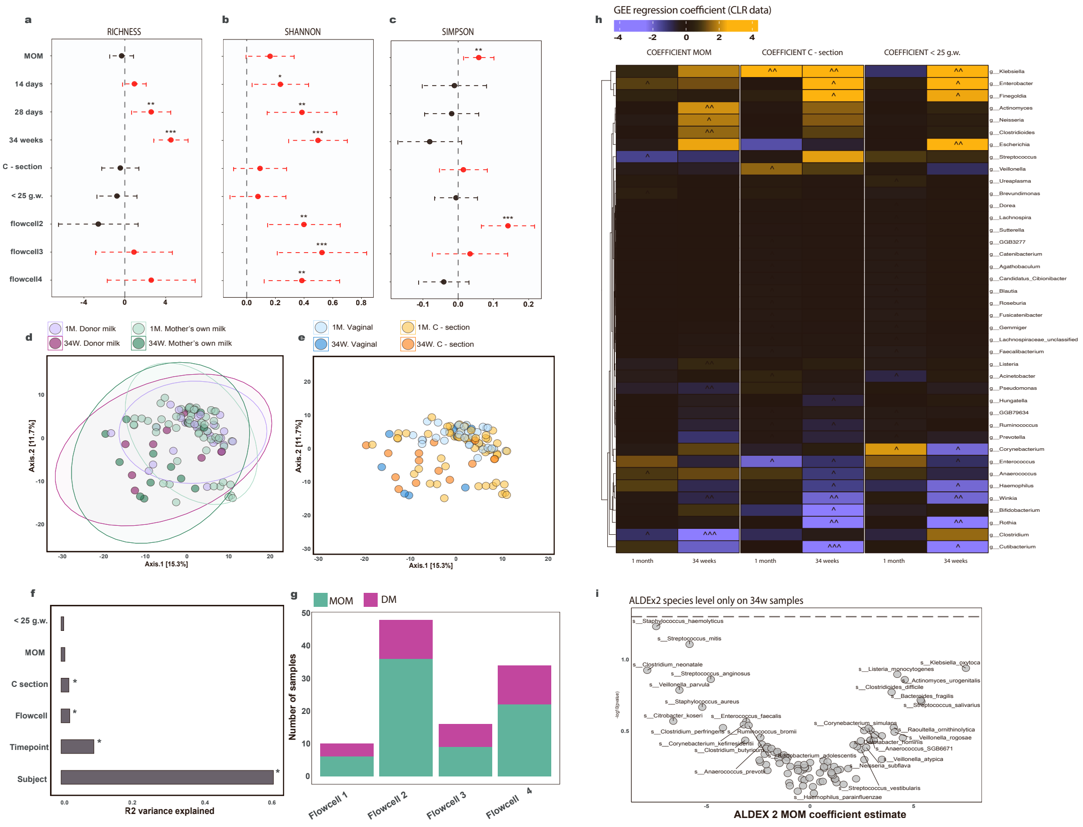

Figure S6. Analysis of gut microbiomes in DM and MOM infants, related to Figure 6. a-c) Coefficient sizes for alpha diversity indices generated with generalized estimating equations (GEE). * p < 0.05, ** p < 0.01, *** p < 0.001. Red color indicates positive association. Black indicates negative association. **d-e**) CLR transformed data plotted in Euclidean space (Aitchison's distance). Samples are colored by timepoint and diet group (d) and mode of delivery (e). Ellipses are drawn at 95% confidence. **f**) R2 variance explained through Permanova analysis with 999 permutations. **g**) Barplot with number of samples on Y-axis and sequencing batch represented by flowcell on X-axis. Color indicates MOM or DM. **h**) Heatmap of differentially abundant taxa at genus level for MOM, C-section and gestational age. Color indicates coefficient sizes from GEE on CLR transformed abundance data. Estimates were generated on 1M or 34 weeks subsetted data. Flowcell was also included as a covariate in the models. Differential abundance was tested with three methods, ALDEx2 on count data, and GEE on CLR and relative abundance data. ^ indicates uncorrected $p < 0.05$ with one method, ^^ two methods, and ^^^ three methods. **i**) Volcano plot showing differentially abundant species in 34-week samples. ALDEx2 MOM coefficient estimates on X-axis and $-\log_{10}(p)$ on Y-axis.

Supplemental Table

Variable	22+4 -24+6 (n=22)	25+1 - 26+6 (n=50)	p-value	Test
Center			0.139	Fisher's Exact Test
Gothenburg, n (%), 11 boys, 11, girls	10 (45)	12 (24)		
Stockholm, n (%), 6 boys, 3 girls	1 (5)	8 (16)		
Lund, n (%), 22 boys, 19 girls	11 (50)	30 (60)		
Mode of delivery, cesarian section, n (%)	8 (36)	33 (66)	**0.019**	Chi-Square Test
Diabetes, n%	0 (0)	0 (0)	-	
Preeclapsia, n%	1 (5)	7 (14)	0.42	Fisher's Exact Test
Parity (including the study subject), n (%)			0.698	Fisher's Exact Test
1	14 (64)	25 (50)		
2	4 (18)	13 (26)		
3	2 (9)	8 (16)		
4	0 (0)	1 (2)		
5	1 (5)	1 (2)		
Plurality , n (%)			0.722	Fisher's Exact Test
1	16 (73)	35 (70)		
2	6 (27)	12 (24)		
3	0 (0)	3 (6)		
Preterm labor, n (%)	20/21 (95)	37/49 (76)	0.056	Fisher's Exact Test
Rupture of the membranes	8/21 (38)	13/49 (27)	0.333	Chi-Square Test
Chorioanionitis	8/21 (38)	18/47 (37)	0.987	Chi-Square Test
Antenatal steroids, n (%)	22 (100)	47/49	1	Fisher's Exact Test
AA:DHA treated, n (%)	10 (45)	23 (46)	0.966	Chi-Square Test
GA (weeks), mean (SD) | media [Q1-Q3]	23.9 (0.7) | 24.0 [23.4-24.4]	26.3 (0.8) | 26.3 [25.6-27.0]	-	
BW (g), mean (SD) | media [Q1-Q3]	628 (100) | 635 [580-687]	883 (163) | 873 [780-965]	**<0.001**	Mann-Whitney U
APGAR at 1 min, mean (SD) | media [Q1-Q3]	4.0 (1.9) | 4 [3-5]	5.7 (2.0) | 6 [5-7]	**<0.001**	Mann-Whitney U
APGAR at 5 min, mean (SD) | media [Q1-Q3]	5.7 (2.2) | 6 [5-7]	7.7 (2.0) | 8 [6-9]	**<0.001**	Mann-Whitney U
APGAR at 10 min, mean (SD) | media [Q1-Q3]	7.6 (2.2) | 8 [6-9]	9.0 (1.1) | 9 [8-10]	**0.007**	Mann-Whitney U
Death, n (%)	2 (9)	1 (2)	0.219	Fisher's Exact Test
At postnatal day	43 and 115	39		
Sepsis, n (%)	6 (27)	12 (24)	0.768	Chi-Square Test
PDA, patent ductus arteriosus, n (%)	3 (13)	18 (36)	**<0.001**	Chi-Square Test
NEC, necrotising enterocolitis, n (%)	3 (14)	4 (8)	0.668	Fisher's Exact Test
BPD, bronchopulmonary dysplasia, n (%)	10/21 (48)	9 (18)	**0.013**	Chi-Square Test
Mechanical ventilation, days, mean (SD) | media [Q1-Q3]	35 (17) | 33 [24-41]	8 (12) | 2 [0-13]	**<0.001**	Mann-Whitney U
Severe Rethinopathy, ROP grade 3 and/or treated), n (%)	10 (45)	5 (10)	**0.001**	Fisher's Exact Test
Severe intraventricular haemorrhage IVH grade 3 or 4), n (%)	3 (14)	5 (10)	0.693	Fisher's Exact Test
Nutrition, first four weeks of life				
Enteral fluids, mL/kg/d, mean (SD) | media [Q1-Q3]	104.2 (28.5) | 108.7 [101.6-121.9]	120.2 (31.2) | 124.8 [104.1-144.6]	**0.01**	Mann-Whitney U
Donor milk, mL/kg/d, mean (SD) | media [Q1-Q3]	24.0 (39.6) | 2.9 [0.5-28.8]	36.5 (45.2) | 14.3 [4.3-59.7]	**0.009**	Mann-Whitney U
Maternal milk, mL/kg/d, mean (SD) | media [Q1-Q3]	76.8 (44.8) | 101.0 [46.9-109.1]	79.9 (49.0) | 89.7 [24.8-119.1]	0.57	Mann-Whitney U
Parenteral fluids, mL/kg/day, mean (SD) | media [Q1-Q3]	60.9 (27.7) | 56.3 [46.6-70.9]	38.3 (28.5) | 28.8 [18.3-50.0]	**<0.001**	Mann-Whitney U
Energy, total, kcal/kg/d, mean (SD) | media [Q1-Q3]	117.0 (10.9) |118.4 [114.0-120.8]	123.3 (11.6) |123.5 [116.2-130.8]	**0.039**	Mann-Whitney U
Protein, total, g/kg/d, mean (SD) | media [Q1-Q3]	3.7 (0.3) | 3.7 [3.5-3.9]	3.7 (0.3) | 3.8 [3.5-3.9]	0.525	Mann-Whitney U
Carbohydrates, g/kg/d, mean (SD) | media [Q1-Q3]	12.6 (0.9) |12.5 [12.3-13.0]	13.1 (0.9) | 13.0 [12.5-13.5]	0.061	Mann-Whitney U
Lipids, total, g/kg/d, mean (SD) | media [Q1-Q3]	5.5 (1.1) | 5.6 [5.0-6.0]	5.9 (1.1) | 6.0 [5.2-6.6]	0.085	Mann-Whitney U
Time to full enteral feeds, days, mean (SD) | media [Q1-Q3]***	21 (9) | 18 [14-26]	14 (6) | 12 [10-15]	**<0.001**	Mann-Whitney U
Parenteral nutrition, days, mean (SD) | media [Q1-Q3]**	30 (18) | 24 [16-37]	14 (6) | 13 [9-19]	**<0.001**	Mann-Whitney U
* First day with enteral fluids > 150 ml/kg/d				
** Any parenteral lipids or aminoacids				

Table S1. Statistical overview of the cohort, related to Figure 1
